# A framework for assessing selection and misclassification bias in mendelian randomisation studies: an illustrative example between body mass index and covid-19

**DOI:** 10.1136/bmj-2022-072148

**Published:** 2023-06-19

**Authors:** Gemma L Clayton, Ana Gonçalves, Soares, Neil Goulding, Maria Carolina Borges, Michael V Holmes, George Davey, Smith, Kate Tilling, Deborah A Lawlor, Alice R Carter

**Affiliations:** 1 MRC Integrative Epidemiology Unit, University of Bristol, Bristol, UK; 2 Population Health Sciences, Bristol Medical School, University of Bristol, Bristol, UK; 3 National Institute for Health Research Bristol Biomedical Research Centre, University Hospitals Bristol NHS Foundation Trust and University of Bristol, Bristol, UK

## Abstract

Mendelian randomisation (MR) studies, which investigate causal effects of exposures on disease, might be biased by sample selection and misclassification if phenotypes are not measured universally with the same definition in all study populations or participants. For example, in MR analyses of effects of exposures on covid-19, studies might include individuals with specific characteristics (eg, high socioeconomic position) meaning they are more likely to be tested for SARS-CoV-2 infection or respond to study questionnaires collecting data on infection and disease (selection bias). Alternatively, studies might assume those who were not tested have not been infected by SARS-CoV-2 or had covid-19 and are included as control participants (misclassification bias). In this article, a set of analyses to investigate the presence of selection or misclassification bias in MR studies is proposed and the implications of these on results is considered. The effect of body mass index on covid-19 susceptibility and severity is used as an illustrative example.

Mendelian randomisation (MR) can be implemented as an instrumental variable analysis, with genetic variants as an instrument for exposures (or modifiable risk factors) to explore their causal effects on the occurrence and prognosis of disease^1^ ^2^ (fig 1). Genetic variants are randomly allocated at conception, and not modified throughout life, meaning MR is less likely to be biased by confounding or reverse causality (ie, confounding by prevalent disease) than conventional multivariable regression.^1^ ^2^ ^3^ In individual level MR (often referred to as one sample MR), the genetic instrumental variable-exposure and genetic instrumental variable-outcome associations are from the same sample, and individual level data are used to derive the MR estimate. In a summary data MR study (often referred to as two sample MR), the genetic instrumental variable-exposure and genetic instrumental variable-outcome associations come from two non-overlapping samples from the same underlying population.^2^ ^4^ We focus on the summary data MR approach in this paper, because this approach is more commonly used in the literature, but all methods described here could be used in an individual level MR analysis. Some biases in MR are discussed frequently in the literature; for example, when the genetic instrument can affect multiple biological pathways not including the trait of interest (genetic horizontal pleiotropy) or when confounding can be caused by the structure of the population (fig 1 and supplementary table 1).^5^ ^6^ ^7^ Bias from selection and misclassification^8^ in MR, however, has been less commonly described or explored, particularly in applied papers.^2^ ^9^ ^10^ ^11^

**Fig 1:**
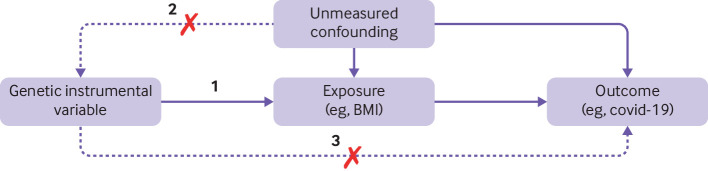
Mendelian randomisation assumptions. Three core assumptions are required for valid causal inference in mendelian randomisation. Genetic instruments must: (1) be robustly associated with the exposure (relevance assumption, illustrated by the arrow from the genetic instrumental variable to the exposure in this directed acyclic graph (DAG); (2) share no common cause with the outcome (independence assumption, illustrated by a dashed arrow with a red cross from confounder to both the genetic instrument and the outcome); and (3) only be associated with the outcome through their effect on the exposure (exclusion restriction assumption, illustrated by the dashed arrow with a red cross from the genetic instrument to the outcome)

## Selection bias

Selection bias can occur when a study sample differs from the target population, which could be caused by non-random participation in the study, subgroup analyses, or missing data, and therefore affects the validity of the study^12^ ^13^ (box 1). When the exposure and outcome (or a cause of these) influence the probability of being selected into the analytical sample, collider bias can be introduced.^16^ In the context of covid-19, selection bias can arise from differential response to covid-19 study questionnaires, differential risk of being exposed to SARS-Cov-2 infection, and differences in who receives a SARS-CoV-2 test or who is admitted to hospital.^14^ ^15^ For example, if a person never receives a SARS-CoV-2 test, their covid-19 status remains unknown, and they would not be selected into any analysis that depends on having a test (table 1). Genome wide association studies (GWAS) are studies where genetic variants are tested for an association with a phenotype (ie, a trait or disease outcome, such as body mass index (BMI)). The most common method for selection of genetic variants to be used as instruments for MR studies is to take variants that associate with the exposure of interest (eg, BMI) at genome wide significance. Selection bias can occur in GWAS when participants selected into the GWAS are a non-random sample of the target population.^18^ ^19^ This non-random selection can result in spurious results in MR analyses when genetic instruments are selected from GWAS affected by this selection bias.

Box 1 **Selection (or collider) and misclassification bias**

Selection bias can occur when a study sample differs from the target population, and as a result affects the validity of the study. Differences from the target population could, for example, be caused by non-random participation, missing data, and loss to follow-up. When the exposure and outcome (or a cause of these) influence the probability of being selected into the analytical sample, collider bias can be introduced. In the presence of selection bias, bias can go in any direction (ie, overestimate or underestimate the true effect).

- In the context of covid-19, selection bias can arise from differential response to covid-19 study questionnaires, differential risk of being exposed to SARS-Cov-2 infection, and differences in who receives a SARS-CoV-2 test or who is admitted to hospital.^14^ ^15^ For example, if a person never receives a SARS-CoV-2 test, their covid-19 status remains unknown, and they would not be selected into any analysis that depends on having a test.

Misclassification occurs when a study participant is incorrectly categorised for a trait or group (ie, when participants who are cases (or in the exposed group) are wrongly classified as controls (or in the unexposed group), or vice versa), and can result in bias in either direction. Misclassification can occur in any variable (ie, exposure, outcome, and confounders).

- Misclassification is particularly likely to be a problem in genome wide association studies of covid-19 susceptibility and severity which included participants when universal testing for SARS-CoV-2 infection was not available. Many countries did not have universal testing, and as the pandemic continues, many countries are stopping universal testing policies. Hence comparing participants with a positive test result for covid-19 with population controls (ie, any person who was not a case of covid-19, including those who had a negative test result or whose SARS-CoV-2 infection status was unknown) could result in misclassification by including asymptomatic or untested people with infection or disease as controls.

**Table 1:** Definitions of cases and controls (from COVID-19 Host Genetics Initiative), the assumed question looked at by comparing risk factors in cases and controls and potential sources of bias induced when used in mendelian randomisation

Group name and date of release*	European individuals in GWAS sample (%)	Contribution of UK Biobank to GWAS sample (%)†	Phenotype		Question looked at by comparing risk factors in cases and controls in relation to BMI	Potential sources of bias in MR between BMI as exposure and each covid-19 outcome definition
			Cases	Controls		
A1 Very severe covid-19 *v* not admitted to hospital for covid-19, release 4 (20 October 2020)	71	0	Very severe respiratory confirmed covid-19 (those admitted to hospital for symptoms associated with laboratory confirmed SARS-CoV-2 infection (RNA or serology based, or both) and required respiratory support, or whose cause of death was associated with covid-19), n=269	Not admitted to hospital for covid-19 (those who had laboratory confirmed SARS-CoV-2 infection (RNA or serology based, or both) and were not admitted to hospital 21 days after the test), n=688	Disease severity: does having a higher average BMI cause an increased chance of admission to hospital (ventilation support or death from covid-19), in those with a positive test result?	Selection bias caused by sample being conditional on having a diagnosis of covid-19 or detected SARS-CoV-2 infection (eg, a positive test result) when factors that predict getting tested or a diagnosis are present.
A2 Very severe covid-19 *v* population, release 6 (15 June 2021)	67	42	Very severe respiratory confirmed covid-19 (those admitted to hospital for symptoms associated with laboratory confirmed SARS-CoV-2 infection (RNA or serology based, or both) and required respiratory support, or whose cause of death was associated with covid-19), n=8779	All population (any person who was not a case, ie, people who were never tested, had a negative test result, or had an unknown testing status), n=1 001 875	Disease severity and susceptibility: does having a higher average BMI cause an increased chance of admission to hospital (ventilation support or death from covid-19)?	Misclassification of cases as controls because control group includes those who were infected with SARS-CoV-2 but have not had a test for covid-19. Selection bias caused by case status being conditional on having a diagnosis of severe covid-19, when factors that predict getting tested or a diagnosis are present
B1 Admitted to hospital for covid-19 *v* not admitted to hospital for covid-19, release 6 (15 June 2021)	76	14	Admitted to hospital for covid-19 (those admitted to hospital for symptoms associated with laboratory confirmed SARS-CoV-2 infection (RNA or serology based, or both)), n=14 480	Not admitted to hospital for covid-19 (those who had laboratory confirmed SARS-CoV-2 infection (RNA or serology based, or both) and were not admitted to hospital 21 days after the test), n=73 191	Disease severity: does having a higher average BMI and a positive test result cause an increased chance of admission to hospital?	Selection bias caused by sample being conditional on having a diagnosis of covid-19, or detected SARS-CoV-2 infection (eg, a positive test result), when multiple factors that predict getting tested or a diagnosis are present.
B2 Admitted to hospital for covid-19 *v* population, release 6 (15 June 2021)	74	21	Admitted to hospital for covid-19 (those admitted to hospital for symptoms associated with laboratory confirmed SARS-CoV-2 infection (RNA or serology based, or both)), n=24 274	All population (any person who was not a case, ie, people who were never tested, had a negative test result, or had an unknown testing status), n=2 061 529	Disease severity and susceptibility: does having a higher average BMI cause an increased chance of admission to hospital, in those with a positive test result?	Misclassification of cases as controls because control group includes those who were infected with SARS-CoV-2 but have not had a test for covid-19.
C1 Positive for covid-19 *v* laboratory or self-reported negative test result, release 4 (20 October 2020)	85	6.5	Positive test result for covid-19 (those who had a laboratory confirmed SARS-CoV-2 infection (RNA or serology based, or both), were clinically diagnosed (ie, electronic health record, ICD coding, or physician confirmed), or self-reported positive test result for covid-19 (eg, via questionnaire)), n=24 057	Laboratory or self-reported negative test result (those who had a negative laboratory SARS-CoV-2 test result (RNA or serology based, or both) or self-reported negative test result for covid-19 (eg, via questionnaire)), n=218 062	Disease susceptibility: does having a higher average BMI cause an increased chance of a positive test result, for those who get tested?	Misclassification of cases as controls because control group includes those with self-reported negative test result for covid-19 but might not have had a test for covid-19 because of selection of who can get a test for SARS-CoV-2 infection or covid-19 disease. Selection bias caused by sample being conditional on having a diagnosis of covid-19 or detected SARS-CoV-2 infection (eg, a positive test result), when factors that predict getting tested or a diagnosis are present
C2 Positive for covid-19 *v* population, release 6 (15 June 2021)	78	17	Positive test result for covid-19 (those who had a laboratory confirmed SARS-CoV-2 infection (RNA or serology based, or both), were clinically diagnosed (ie, electronic health record, ICD coding, or physician confirmed), or self-reported positive test result for covid-19 (eg, via questionnaire)), n=112 612	All population (any person who was not a case, ie, people who were never tested, had a negative test result, or had an unknown testing status), n=2 474 079	Disease susceptibility: does having a higher average BMI cause an increased chance of a positive test result?	Misclassification of cases as controls because comparison includes those who have not had a test for covid-19 and assumed to be a control
D1 Predicted covid-19 *v* predicted or self-reported negative test result, European release 4 (20 October 2020)	100	0	Predicted covid-19 from self-reported symptoms (those with a value from the predictive model >−0.44 or self-reported covid-19 positive test result)‡ n=3204	Predicted or self-reported non-covid-19 (those with the minimum possible value from the predictive model (ie, 1.32) and not self-reported positive test result for covid-19)‡ n=35 728	Disease susceptibility; does having a higher average BMI cause an increased chance of having covid-19, for those who have symptoms?	Misclassification bias in case and control groups caused by misreporting of symptoms and unknown infection status

Those “admitted”/“not admitted” to hospital are referred to by the COVID-HGI naming convention as “hospitalised”/“not hospitalised”. Index event bias caused by conditioning on index infection will affect those either admitted to hospital or diagnosed with very severe covid-19 (eg, A1, A2, B1, B2). Survival bias caused by being alive long enough to be included in case or control definitions will affect all comparisons

GWAS=genome wide association studies; BMI=body mass index; MR=mendelian randomisation; ICD=international classification of diseases, 10th revision.

* Release of data refers to each round of the GWAS meta-analyses conducted and made available by the COVID-19 Host Genetics Initiative, where not all case or control definitions were included in every meta-analysis or release of data.

† Proportion of individuals in the COVID-19 Host Genetics Initiative GWAS who were from UK Biobank. UK Biobank contributed to both BMI and covid-19 GWAS, where sample overlap between exposure and outcome GWAS in summary data mendelian randomisation can introduce bias (see Methods and results for primary analysis).

‡ Prediction based on Menni et al.^17^

## Misclassification bias

Measurement error is the difference between a measured quantity and its true value. In MR studies, non-differential measurement error in the exposure can lead to weak instrument bias (when the relation between the genetic instrumental variables and the risk factor or exposure is small in magnitude or imprecise, or both^20^ ^21^) and, in the outcome, imprecision of estimates, whereas differential measurement error can bias observed estimates towards or away from the null^10^ ^22^ (box 1). Misclassification occurs when a study participant is incorrectly categorised for a trait or group^10^ (ie, when cases are wrongly classified as controls, or vice versa) and can result in bias in either direction,^10^ ^22^ and can occur in any variable (ie, exposure, outcome, and confounders). Misclassification is particularly likely to be an issue in GWAS of covid-19 susceptibility and severity which included participants when universal testing for SARS-CoV-2 infection was not available. Many countries did not have universal testing and, as the pandemic continues, many countries are stopping universal testing policies. Hence comparing participants with a positive test result for covid-19 with population controls (ie, any person who was not a case, including those who had a negative test result or whose SARS-CoV-2 infection status was unknown) could result in misclassification by including asymptomatic or untested people with infection or disease as controls (table 1). This approach would lead to misclassification bias and could result in a violation of the MR exclusion-restriction criteria (fig 1).^11^

**Summary points**

Mendelian randomisation (MR) studies use genetic variants randomly allocated at conception as instruments to test potential effects of exposures on disease
Although MR studies are typically not biased by confounding factors that influence conventional observational analyses (eg, socioeconomic position) and reverse causality, non-representative sample selection or misclassification could bias MR studies of highly selected phenotypes, such as covid-19
A range of methods and approaches are proposed, such as using different definitions for cases and controls, a no relevance control, testing for genetic correlation, and adjusting for predictors of selection to investigate selection or misclassification bias, or both, in MR of selected phenotypes
A framework of analyses is provided for exploring selection and misclassification bias in MR studies and guidance on how the results of these analyses can be interpreted

## Illustrative example

The covid-19 pandemic was declared on 11 March 2020.^23^ As well as the increased call to understand the spread of SARS-CoV-2 infection, identifying individuals at increased risk of becoming infected with SARS-CoV-2 and who might subsequently develop covid-19 and severe disease was important. BMI became an exposure of interest. Along with studies with traditional epidemiological designs (ie, multivariable analyses with observed phenotypic data) to explore BMI and covid-19 susceptibility and severity, an increasing number of MR studies were conducted with the aim of determining causality.^24^ ^25^ ^26^ ^27^ ^28^ ^29^ ^30^ These studies (summarised in supplementary table 2) mostly concluded a causal effect of genetically predicted higher BMI on increased risk of covid-19 susceptibility and severity. Most of these studies used population controls, and other than being described as a limitation in some, few attempts were made to explore selection and misclassification bias.

### Data

We used summary data from the 2018 Genetic Investigation of Anthropometric Traits (GIANT) Consortium from individuals of predominantly European descent to genetically instrument BMI.^31^ Summary data on the association of single nucleotide polymorphisms (SNPs) for covid-19 susceptibility and severity were obtained from the covid-19 host genetics initiative (COVID-19 HGI).^32^ ^33^ This initiative includes 162 contributing studies worldwide, predominantly from Europe and the US. We used all case and control comparison groups analysed by the GWAS consortium (table 1 and see Comparison of different case and control groups). For consistency with the COVID-19 HGI definition, here we refer to cases as individuals who had covid-19 (our outcome of interest) and controls as individuals who did not have covid-19.

### Methods and results for primary analysis

**Fig 2:**
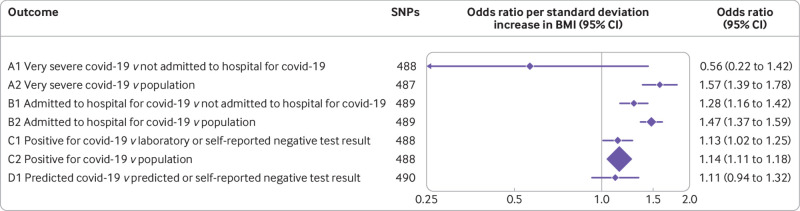
Main analysis: effect of body mass index (BMI) on SARS-CoV-2/covid-19. CI=confidence interval; SNPs=single nucleotide polymorphisms

Our primary applied analysis (assuming no selection or misclassification bias) used inverse variance weighting with multiplicative random effects to estimate the effect of BMI on covid-19 susceptibility and severity.^34^ In inverse variance weighted estimates, we found that genetically predicted higher BMI was associated with a higher odds of covid-19 for all groups, except A1 (very severe covid-19 *v* not admitted to hospital for covid-19, odds ratio 0.56 per 1 standard deviation higher BMI, 95% confidence interval 0.22 to 1.41) which had the smallest sample size (n=269 cases, n=688 controls). For all other case and control comparisons, odds ratios ranged from 1.14 (95% confidence interval 1.11 to 1.18) for group C2 (positive for covid-19 *v* population) to 1.57 (1.39 to 1.78) for group A2 (very severe covid-19 *v* population) (fig 2). 

We conducted a series of sensitivity analyses to assess the validity of the core MR assumptions (more details here^1^ ^2^ ^3^). We estimated the mean F statistics and total R^2^ (to check the relevance assumption and weak instrument bias), used MR-Egger^34^ and weighted median^35^ methods (to assess horizontal pleiotropy), used skin tanning as a negative control outcome (to assess population stratification), and repeated the analysis excluding the UK Biobank in the exposure GWAS (to assess bias from sample overlap). The results of these analyses suggested that the core MR assumptions were plausible. All methods and results are described in the supplementary material (supplementary tables 1-6 and supplementary figs 1-7). We interpreted the results under the monotonicity assumption, which assumes the direction of effect on the exposure from varying the level of the instrumental variable should be in the same direction for all individuals (for more information see Dixon et al^36^). For example, for A1, this corresponds to the average effect of BMI on very severe covid-19 for individuals whose BMI was affected by the 488 BMI increasing SNPs (supplementary fig 2). 

## Approaches to explore potential selection and misclassification bias

**Fig 3:**
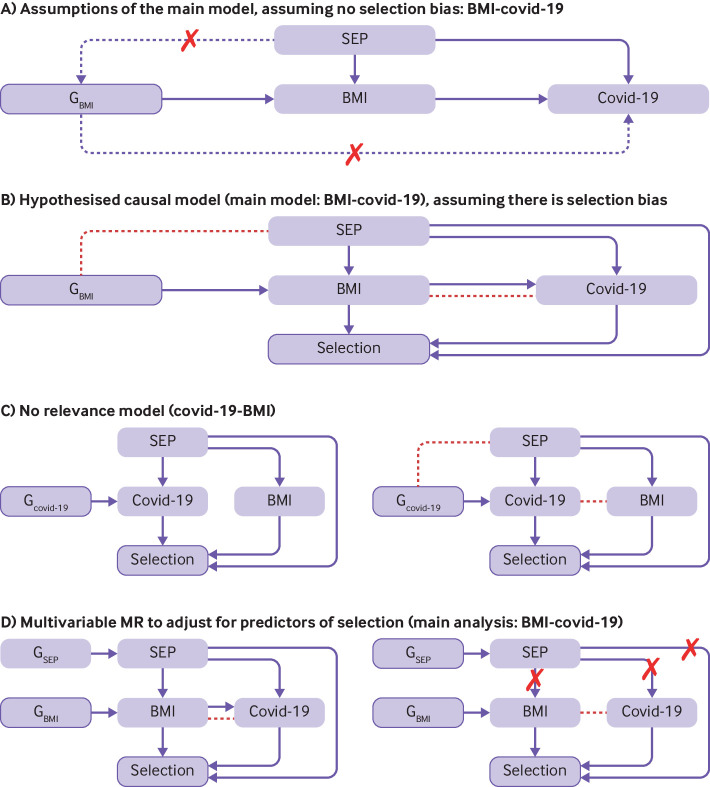
Casual assumptions made for methods to assess selection or misclassification bias with directed acyclic graphs (DAGs), where socioeconomic position (SEP) is used as an example of a confounder and predictor of selection. (A) The assumption is that the generic variants for body mass index (BMI) must: be robustly associated with BMI (relevance assumption, illustrated by a solid arrow from the genetic instrumental variable to the exposure; not be associated with any confounder (here, SEP) between the exposure and outcome (independence assumption, illustrated by a dashed arrow line with a red cross from confounder to both the genetic instrument and the outcome); and only be associated with the outcome (covid-19) through their effect on the exposure (BMI) (exclusion restriction assumption, illustrated by the dashed arrow with a red cross from the genetic instrument to the outcome). (B) Exposure (BMI), outcome (covid-19), and confounder (SEP) affect selection (indicated by solid arrow between BMI and covid-19) and the confounder (SEP) also affects the exposure and outcome. In this case, selection is a collider, and is implicitly conditioned on in analyses (indicated by outlined purple box), which can induce a spurious association between BMI and covid-19 (red dashed line). The solid directional arrow is thought to be a true causal effect and the red dashed line the induced bias. (C, left) In the no relevance model, the assumption is that the genetic variants for covid-19 must: be robustly associated with covid-19; not be associated with SEP; and not be associated with BMI through their effect on covid-19. (C, right) An effect of covid-19 on BMI (a spurious association, red dashed line) would indicate potential bias (selection, misclassification, or horizontal pleiotropy) in the main analysis model (BMI-covid-19). (D, left) As well as the assumptions in (B), another assumption is that the genetic variants for BMI and SEP are independent, and that SEP fully predicts selection. (D, right) By adjusting for SEP, the back door pathway is closed between SEP-BMI, SEP-covid-19, and SEP-selection (indicated by red crosses). Therefore, by adjusting for SEP as a predictor of selection, bias is removed in the BMI-covid-19 association through SEP only. Bias due to BMI and covid-19 causing selection is still present. G_BMI_=genetic variant for BMI; G_covid-19_=genetic variant for covid-19; G_SEP_=genetic variant for SEP; MR=mendelian randomisation

We outline different approaches that can be used in a summary data MR analysis to explore the potential presence of selection or misclassification bias, or both. The methods proposed here are not new but rather the application of them together to assess selection or misclassification bias in MR studies is new. We have combined selection and misclassification bias because in real scenarios we often do not know how the two sources of bias work together, but we suspect they do (eg, sample selection can lead to misclassification); the methods proposed here could be applied to either bias. We describe these approaches, including the concept and rationale, apply these methods to our illustrative example (BMI-covid-19), and compare our results with the primary analysis (inverse variance weighted not taking account of these potential biases), and then refer to other examples where these methods are likely to be important. Figure 3 shows how bias can be induced and how the methods proposed can be used to assess bias. Box 2 provides an overview of each method.

Box 2 **Summary of mendelian randomisation methods carried out to investigate selection and misclassification bias in analyses of body mass index and covid-19 susceptibility and severity**

**Different case and control definitions**

Mendelian randomisation (MR) studies almost always rely on genome wide association studies (GWAS) to select genetic instruments for the exposure of interest. GWAS can be biased by misclassification of the phenotype and by selection of who is included in the GWAS. Examining the consistency of effects across different definitions of cases and controls allows for assessment of how different sources of misclassification or selection bias might introduce error. When results are consistent across definitions, the evidence for a causal effect is greater. When effect estimates diverge across different definitions, however, the evidence might suggest that selection or misclassification bias is present and further analyses or exploration is needed to understand which, if any, of the different definitions of cases and controls used in a GWAS is unbiased.

**No relevance study**

MR no relevance analyses are carried out in a subpopulation or the same population where the assumption is made that the exposure of interest cannot plausibly influence the outcome, and hence the genetic instrument is not expected to relate to the outcome of interest. No relevance studies have previously been used in MR analyses to test for bias caused by horizontal pleiotropy.^37^ These studies can also be used to test for selection bias, with the same principle that the exposure is not a plausible cause of the outcome and that sources of selection in the no relevance analyses are similar to those in the real study. Under these assumptions, if a non-null association is seen in the no relevance study, it suggests that the results in the main study might be explained by selection bias (or other sources of bias).

**Genetic correlation**

If a correlation between genetic variants related to an exposure of interest and traits that predict selection exists, such as socioeconomic position and related behaviours, this correlation indicates possible selection bias in the GWAS for the exposure of interest. Specifically, some genetic variants might spuriously associate with the exposure of interest because of its genetic correlation with selection factors. Testing for correlation of single nucleotide polymorphisms between exposure or outcome, and hypothesised predictors of selection identifies whether the GWAS might be associated with the phenotype of interest, or whether the GWAS are identifying factors associated with selection.

**Multivariable mendelian randomisation**

Multivariable MR can estimate a potential causal effect adjusted for factors that predict selection. Adjusting for factors that are associated with selection in conventional observational analyses has been appreciated and used for many decades^16^ but so far, this approach has not been used widely in MR. After genetic correlation analyses, where a correlation is observed with predictors of selection, multivariable MR can be used to control for selection bias. As in conventional multivariable regression, however, there are situations where adjusting the main MR analyses for some predictors of selection can induce bias. This situation is particularly true when the selection predictors are mediators or colliders, or both, of the potential effect of exposure on outcome.^38^ ^39^ In conventional multivariable regression analyses, this problem of colliders can be resolved in some situations by using methods such as multiple imputation that separate and control for selection (eg, variables related to missing data) analyses from the main analysis of exposure-outcome effects. These methods are not currently available for GWAS and MR. Therefore, knowledge of the subject matter is important to determine which mechanism would be most likely, and what variables might be predictors of selection in such an analysis require careful consideration.

**Comparisons over time**

When changes to testing, classification, or diagnosis exist, which could result in changes in those diagnosed with or without the disease of interest and therefore a change in the sample used in GWAS, analyses should be repeated. These analyses can be done separately (as was done here) or with recently proposed methods in a time varying framework.^40^

Note that these methods are not explicitly for testing for selection or misclassification bias and can be used to test for different sources of bias in MR.

### Comparison of different case and control groups

The availability of different sources of data (eg, mortality records, hospital records, and testing data) or case definitions (eg, disease incidence *v* disease severity or different diagnostic criteria) means different case and control definitions can be defined and therefore different causal questions answered. When these definitions are affected differentially by selection or misclassification bias, results can be compared to evaluate the potential effect of such biases.

In our illustrative example, we compared different definitions of cases and controls provided by COVID-19 HGI to explore selection and misclassification bias. In brief, the case and control comparisons used were:

- Very severe respiratory confirmed covid-19 versus not admitted to hospital for covid-19 (A1)
- Very severe respiratory confirmed covid-19 versus population (ie, individuals from population based cohorts, including individuals whose SARS-CoV-2 infection status was unknown or who had a negative test result) (A2)
- Admitted to hospital for covid-19 versus not admitted to hospital for covid-19 (B1)
- Admitted to hospital for covid-19 versus population (B2)
- Positive result for covid-19 (either test or self-report) versus laboratory or self-reported negative test result (C1)
- Positive result for covid-19 versus population (C2)
- Predicted covid-19 from self-reported symptoms versus predicted or self-reported non-covid-19 (D1).

When the case definitions were the same (eg, positive for covid-19 in C1 and C2), only those tested for SARS-CoV-2 (or those who self-reported a positive test result for covid-19) could be included as cases, and selection bias is possible because of individual characteristics related to having a test (in relation to cases) or similarly self-reporting a negative test result for covid-19 (in relation to controls), and therefore being included in the analysis. The control groups for C1 and C2 differed, and the use of population controls in C2 could lead to differential misclassification (eg, misclassification of cases as controls because comparison includes those who have not had a test for covid-19 and assumed to be a control). If effect estimates are similar across different control definitions, selection or misclassification bias, or both, might not be present. If the point estimates differ, this could indicate that selection or misclassification bias, or both, is present. Table 1 and box 2 provide more details.

In our example, the effect estimates for the association between BMI and covid-19 were consistent across all comparisons except for A1 (fig 2, and supplementary fig 3(a)). This finding might be because this comparison group is at the most risk of selection bias because very severe respiratory confirmed covid-19 depends on both getting a test and being admitted to hospital, or because it has a much smaller sample size and is therefore imprecise. We used the latest data from COVID-19 HGI (release 6, 15 June 2021) for the main analyses. Not all outcomes were included in this release, however, and we used the next most recent data for these (groups A1, C1, and D1). In October 2020, COVID-19 HGI recommended that groups A1, C1, and D1 should not be considered (no further explanations were given on the website), but we used these groups in this study because the aim was to assess selection and misclassification bias, and the different definitions of cases and controls can be informative.

Comparing results from different groups of cases and controls is likely to be useful in MR studies of any disease with no, or only non-specific, symptoms and no universal testing. Clinical diagnoses for these diseases are influenced by factors related to whether people see a healthcare professional and, when they do, whether that professional investigates the disease. Examples include type 2 diabetes, gestational diabetes, depression, dysmenorrhoea, and hypertension. Unlike the COVID-19 HGI GWAS, most consortia of GWAS combine different definitions of cases and controls to increase power, meaning that comparing across different case-control groups in MR is not possible. But more recently, attempts have been made to explore the effect of different case-control definitions on GWAS results. For example, in a GWAS of gestational diabetes,^41^ MR-MEGA^42^ (Meta-Regression of Multi-Ethnic Genetic Association) was used to explore whether case definitions (universal blood based testing in all women *v* clinical decision or screening for risk factors) contributed to heterogeneity between studies in the GWAS results, and found that it did not, although the authors acknowledged that limited power might mean important heterogeneity could have been missed.^43^ As the available data for many GWAS become larger, providing results grouped by different definitions of cases and controls will be useful so that MR results can be compared across these comparisons.

### No relevance control analysis

A no relevance study is a form of negative control analysis where analyses are carried out in a subpopulation of the real or relevant study population, where the exposure of interest could not plausibly influence the outcome, and hence the genetic instrument is not expected to relate to the exposure of interest.^44^ This approach has previously been used in MR studies to assess bias caused by horizontal pleiotropy.^37^ ^45^ ^46^ For example, in people who have never smoked, genetic variants that relate to the intensity of cigarette smoking would not be expected to relate to an outcome (eg, cardiovascular disease) other than from bias, such as unbalanced horizontal pleiotropy. Thus an interaction between variants related to the intensity of smoking and ever smoking can be used to determine causality, as shown in a study of the potential effect of maternal gestational smoking on fetal growth parameters.^46^ In that study, a causal effect of smoking on fetal growth was supported by seeing effects only (or stronger effects) in those who had ever smoked cigarettes, whereas seeing similar associations in those who had never smoked would be interpreted as evidence that an apparent MR causal effect is spurious. As recently discussed,^47^ however, stratifying on subgroups can induce collider bias. Researchers should be conscious of potentially inducing other forms of bias when trying to account for selection or misclassification bias. Similarly, no relevance studies can be used to explore selection or misclassification bias when there is no hypothesised true causal effect between exposure and outcome, other than by sample selection (fig 3C). Any evidence of an association might therefore indicate that selection, misclassification, or other biases of concern are present.

In the context of covid-19, and any other new human infectious diseases, it is implausible that the diseases could influence outcomes that existed before the new infectious disease emerged. Thus we used covid-19 as the exposure and BMI as the outcome in a no relevance study to determine whether genetically instrumented covid-19 was related to BMI (assessed before covid-19 existed). In the absence of biases, we would expect null findings. If any effects of covid-19 on BMI are seen, this suggests the presence of selection or misclassification bias, or both, or that other biases, such as horizontal pleiotropy, might bias the main (BMI-covid-19) analysis.

**Fig 4:**
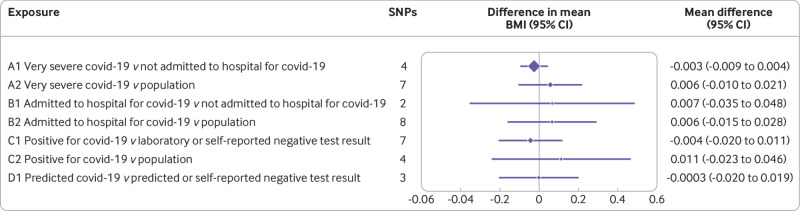
No relevance control analysis: effect of SARS-CoV-2/covid-19 on body mass index (BMI). Mean difference in BMI (standard deviation) comparing genetic liability to covid-19 covers a narrow range, from −0.06 to 0.06. For example, for A1 and C2, the mean difference in BMI (standard deviation) per unit change in log(odds) of covid-19 was −0.003, 95% confidence interval −0.009 to 0.004 and 0.011, −0.023 to 0.046, respectively. CI=confidence interval; SNPs=single nucleotide polymorphisms

For all seven groups of cases and controls in the no relevance analyses, the effect estimates of genetic liability to covid-19 on BMI measured before the pandemic were close to the null, suggesting little evidence of an effect (fig 4). Noteworthy is that Figure 4 covers a narrow range, from −0.06 to 0.06, representing the mean difference in BMI (standard deviation) comparing genetic liability to covid-19. For example, for A1 and C2, the mean difference in BMI (standard deviation) per unit change in log(odds) of covid-19 was −0.003, 95% confidence interval −0.009 to 0.004 and 0.011, −0.023 to 0.046, respectively. The results were consistent when MR-Egger and weighted median methods were used (supplementary fig 3(b)). The only two exceptions were for groups A1 and D1, when MR-Egger suggested a positive effect of genetic liability to covid-19 on BMI. Some evidence exists of heterogeneity between SNPs in groups A1, A2, and B2 for the associations between genetic liability to covid-19 and BMI (supplementary table 4 and supplementary fig 6) and some evidence of horizontal pleotropic effects in group A1 (supplementary table 5).

The results of the no relevance analysis might have been biased by weak instruments because of far fewer SNPs for any covid-19 than for BMI (the exposure in our real illustrative example). The F statistics for the no relevance covid-19 instruments ranged from 22 to 134 (supplementary table 6), however, suggesting weak instrument bias was unlikely. Our no relevance analysis is only valid if our assumption that liability to covid-19 could not influence outcomes such as BMI that were assessed before covid-19 existed. Susceptibility to covid-19 is likely influenced by immune responses against infections, however, which were present before BMI was assessed. Previous analyses exploring the association between covid-19 and stroke have been framed in this way.^48^ Because covid-19, like other diseases, is a binary trait, these estimates represent the liability to covid-19.^49^ This interpretation should be considered by researchers conducting a no relevance analysis with a binary exposure.^49^ ^50^

We recommend that a no relevance test of bias could be used in other MR analyses as part of a collection of sensitivity analyses. For example, when concerns exist about potential selection bias in MR of disease progression,^51^ finding effects of liability to covid-19 on disease progression, particularly for progression outcomes that were assessed in genetic studies before covid-19 existed, could contribute to exploring this bias.

A complementary approach could be positive controls. For example, if concerns exist about MR analyses where selection bias might be a problem (eg, MR of a new biomarker for cardiovascular diseases in a study where selection in those with blood samples is present), comparing MR results (of the new biomarker) with an established blood based causal exposure (eg, low density lipoprotein cholesterol with cardiovascular disease) would be valuable. If the results for the established causal factor are consistent with the existing literature for its effect, this consistency would suggest that selection for having a blood test is not markedly influencing the results.

### Genetic correlation

Identifying genetic correlation between the disease phenotype of interest and any hypothesised predictors of selection,^52^ such as socioeconomic position and associated health behaviours, can be informative in understanding whether selection bias is present. When genetic correlation between the disease phenotype and traits that predict selection is high, this might indicate that GWAS for the disease phenotype are identifying factors associated with getting a test for disease, rather than the disease itself. This situation can be investigated with summary or individual participant GWAS data with linkage disequilibrium score regression.

We estimated the genetic correlation between covid-19 SNPs and SNPs associated with potential predictors of testing (smoking, education, and income),^53^ determined a priori from estimates in previous studies^14^ ^15^ ^54^ and available GWAS summary data. We found evidence of genetic correlation between most covid-19 outcomes and BMI, smoking, education, and income in three of the covid-19 GWAS (but not for C1 and D1 GWAS) (table 2). The correlations ranged from -0.68 for C1 with education to 0.35 for A2 with BMI. The genetic correlation with each predictor of getting tested (selection) was overall similar across the different case and control comparison groups. This similarity suggests that summary statistics for covid-19 might also be identifying genetic predictors of selection. However, it is plausible that hypothesised predictors of selection also have a true association with covid-19 susceptibility and severity, and therefore the need to obtain a test. For example, individuals with lower levels of educational attainment, a measure of socioeconomic position, might be unable to work from home and are therefore exposed to the SARS-CoV-2 virus at greater levels than those with higher educational attainment. Predictors of selection could be explored with multivariable MR where summary data for the selection factors are available (see Multivariable MR adjusting for potential predictors of selection).

**Table 2:** Linkage disequilibrium score regression of genetic correlation between SARS-CoV-2 or covid-19 comparisons groups, body mass index, and possible predictors of selection

Outcome	BMI			Smoking			Education			Income	
	rG (SE)	P value		rG (SE)	P value		rG (SE)	P value		rG (SE)	P value
A1 Very severe covid-19 *v* not admitted to hospital for covid-19*	NA	—		NA	—		NA	—		NA	—
A2 Very severe covid-19 *v* population	0.35 (0.05)	<0.001		0.23 (0.08)	0.003		−0.24 (0.04)	<0.001		−0.27 (0.06)	<0.001
B1 Admitted to hospital for covid-19 *v* not admitted to hospital for covid-19	0.30 (0.06)	<0.001		0.19 (0.08)	0.027		−0.20 (0.05)	<0.001		−0.20 (0.06)	0.002
B2 Admitted to hospital for covid-19 *v* population	0.35 (0.04)	<0.001		0.25 (0.06)	<0.001		−0.33 (0.04)	<0.001		−0.26 (0.05)	<0.001
C1 Positive for covid-19 *v* laboratory or self-reported negative test result	NA	—		0.13 (0.44)	0.763		−0.68 (0.75)	0.364		−0.26 (0.82)	0.752
C2 Positive for covid-19 *v* population	0.32 (0.04)	<0.001		0.13 (0.07)	0.062		−0.34 (0.04)	<0.001		−0.19 (0.05)	<0.001
D1 Predicted covid-19 *v* predicted or self-reported negative test result	0.10 (0.09)	0.268		0.20 (0.21)	0.334		−0.02 (0.07)	0.757		−0.19 (0.11)	0.070
BMI	—	—		0.28 (0.04)	<0.001		−0.27 (0.01)	<0.001		−0.22 (0.02)	<0.001

BMI=body mass index; rG=genetic correlation between two traits; SE=standard error; NA=not applicable.

* Small heritability for this phenotype and therefore genetic correlation cannot be estimate.

Linkage disequilibrium score regression was developed to estimate genetic correlation between traits, partly as a method to quantify the effect of potential confounders. Where two traits (eg, BMI and education) are identified to be genetically correlated, an assumption is made that one causes the other (or vice versa) or that a third (confounding) factor causes them both. In this case, we assumed that a third variable, selection, was associated with the two traits (eg, BMI and education). As with covid-19, linkage disequilibrium score regression could be used to assess potential selection bias in other highly selected phenotypes where diagnostic or screening tests are not universal, such as screening for type 2 diabetes, gestational diabetes, and prostate cancer. Observational studies have shown that risk factors for prostate cancer are the same risk factors for prostate specific antigen testing,^55^ ^56^ suggesting that the risk factors for prostate cancer might be partially caused by selection bias.

### Multivariable MR adjusting for potential predictors of selection

Multivariable MR estimates the causal effects of exposures with adjustment for covariables, as in multivariable regression.^38^ This method requires that the exposure of interest and anything that we want to adjust for have complete GWAS summary data available and valid separable instruments that are strong (conditional on the other instruments and exposures). Multivariable MR can be used to assess the presence of horizontal pleiotropy, mediation of the exposure of interest by another factor, or selection or misclassification bias, as in this example.^57^ ^58^ The approach of adjusting for predictors of selection has previously been described in the non-MR literature as a method to account for selection bias, and has more recently been extended to the MR setting.^16^ ^57^ With the same hypothesised predictors of selection as in linkage disequilibrium score regression, we used multivariable MR to adjust for these variables to estimate the effect of BMI on covid-19, independent of potential influences of receiving a test for SARS-CoV-2 infection 38 (fig 3D). An effect that is different to the effect in the main analysis could indicate the potential presence of selection bias. We used summary statistics from the most recent GWAS for the potential predictors of selection smoking, education, and income (supplementary table 7).

**Fig 5:**
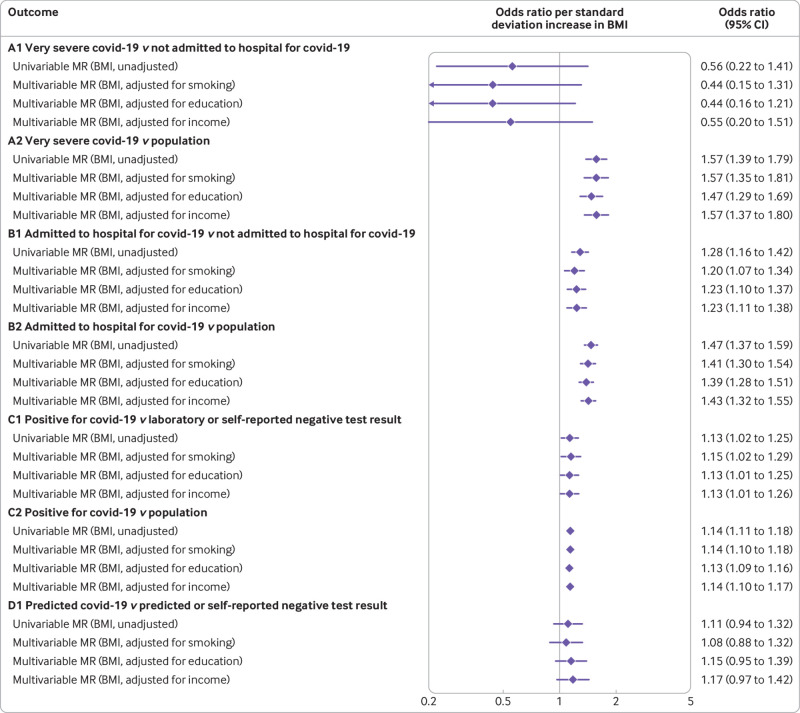
Multivariable mendelian randomisation: effect of body mass index on SARS-CoV-2/covid-19 adjusted for education (years of schooling, dataset ieu-a-1239 https://gwas.mrcieu.ac.uk/datasets/ieu-a-1239/), smoking (cigarettes per day, dataset ieu-b-25 https://gwas.mrcieu.ac.uk/datasets/ieu-b-25/), and income (dataset ukb-b-7408 https://gwas.mrcieu.ac.uk/datasets/ukb-b-7408/). All estimates based on inverse variance weighted models. BMI=body mass index; CI=confidence interval

Results from multivariable MR, adjusted for smoking, education, and income were similar to the main analysis (fig 5), suggesting that the results were not biased by these hypothesised predictors of selection. For example, for the association between BMI and the outcome admitted to hospital for covid-19 versus not admitted to hospital for covid-19, the odds ratio was 1.28, 95% confidence interval 1.16 to 1.42 in the main analysis compared with 1.20, 1.07 to 1.34 when accounting for smoking.

Similar to approaches for assessing selection bias in multivariable regression and other statistical analyses, there are untestable assumptions in multivariable MR. These assumptions include that we know all of the predictors of selection (ie, the selection mechanism), and crucially that we can instrument these selection factors. Both of these assumptions depend on knowledge of the subject matter and data availability. Also, if BMI (ie, the exposure) or covid-19 (ie, the outcome) itself affects selection (fig 3D), then adjusting for predictors of selection will not attenuate all bias. Adjusting for predictors of selection removes the part of bias that acts through these predictors only. Moreover, if the predictors of selection are mediators for the effect of the exposure on the outcome, then we do not want to adjust for it. Although we did not find evidence of an attenuated effect in our example, if the result from multivariable MR adjusting for potential predictors of selection was attenuated compared with the unadjusted effect, this result could indicate selection bias or other explanations (eg, presence of a pleiotropic pathway or because of adjusting for a mediator).^59^ To be able to distinguish between these different explanations is important because otherwise it might be inferred incorrectly that the correct effect is the attenuated one, and the attenuated result reflects removal of some of the real effect (ie, removal of mediating paths). Therefore, researchers need to use knowledge of the subject matter to determine which would be most likely and carefully consider whether predictors of selection are also likely to be mediators of the main effect that we would not want to adjust for. Moreover, although not specifically for selection bias, adjusting for common causes of survival and the outcome has more recently been proposed as a method to account for survival bias in MR.^60^

We adjusted for each selection trait individually, but if evidence exists that each individual variable contributed to selection, adjustment for all traits simultaneously could be done to consider their joint contribution. This approach would require more checks for conditional instrument strength. In our results, we found insufficient evidence that any of these traits individually were drivers of selection bias because we found little difference in the effect estimates from the results of the main MR and the multivariable MR. Therefore, because these methods are relatively underpowered, we felt that conducting a model adjusted for all traits simultaneously was not necessary because it would be difficult to meaningfully interpret any differences in effect estimates.

### Comparisons over time

Where selection pressures have changed over time, a time varying approach to MR can be useful for exploring the effect of selection bias.^40^ ^61^ For covid-19, selection pressures changed as pandemic control measures changed; for example, the selective testing at the start (eg, symptomatic cases only) became almost universal, and then widespread testing came to an end in many places.

Because more studies and more participants were included in more recent releases of the COVID-19 HGI GWAS, we repeated the primary inverse variance weighted method and the no relevance analyses for two of the comparison groups. We used the outcomes admitted to hospital for covid-19 versus not admitted to hospital for covid-19 (B1) and positive for covid-19 versus population (C2) with different releases of the COVID-19 HGI GWAS data, covering time periods up to July 2020 (release 3), October 2020 (release 4), January 2021 (release 5), and June 2021 (release 6). For the other groups, repeat releases of GWAS summary data across all time points were not available.

**Fig 6:**
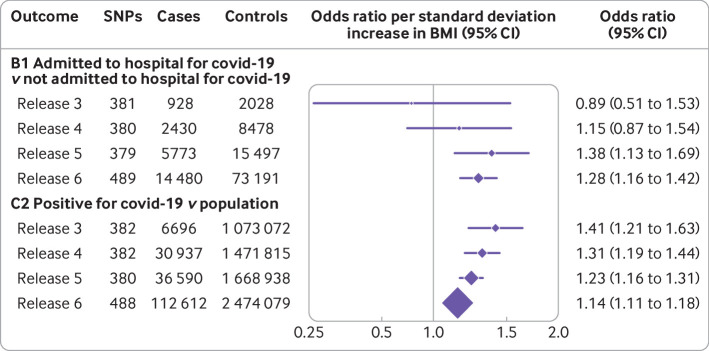
Main analysis: effect of body mass index (BMI) on SARS-CoV-2/covid-19 by version of COVID-19 Host Genetic Initiative release date. Odds ratio corresponds to inverse variance weighing estimates. Version 3 was released on 2 July 2020, version 4 on 20 October 2020, version 5 on 18 January 2021, and version 6 on 15 June 2021. CI=confidence interval; SNPs=single nucleotide polymorphisms

When considering how the main analyses had changed over time, we found that the point estimate for the odds ratio between BMI and severity of covid-19 (group B1) increased over the course of the pandemic (odds ratio 0.89, 95% confidence interval 0.51 to 1.53 based on data from release 3 compared with 1.28, 1.16 to 1.42 based on data from release 6) (fig 6), although confidence intervals between the time points overlapped, reflecting differences in power and precision. Meanwhile, the point estimate for the odds ratio between BMI and covid-19 susceptibility (group C2) decreased over time, but we found evidence of an association at all time points (odds ratio 1.41, 1.21 to 1.63 from release 3 compared with 1.14, 1.11 to 1.18 from release 6).

**Fig 7:**
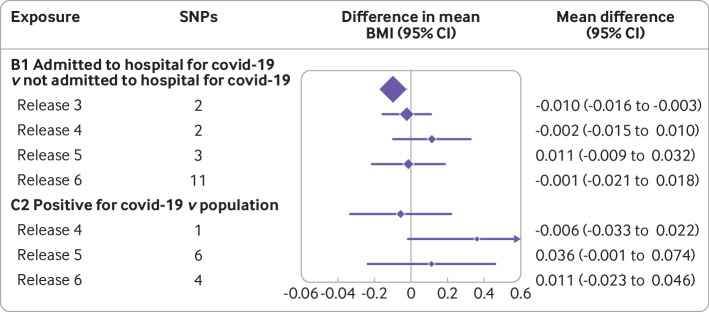
No relevance analysis: effect of SARS-CoV-2/covid-19 on body mass index (BMI) by version of COVID-19 Host Genetic Initiative release date. Version 3 was released on 2 July 2020, version 4 on 20 October 2020, version 5 on 18 January 2021, and version 6 on 15 June 2021. For comparison group B1, a threshold of P<5×10^-6^ was used for selection of single nucleotide polymorphisms (SNPs) (across all releases to allow for comparison) because of the inexistence or small number of SNPs associated at the conventional significant level for the genome wide association studies. No SNPs were available for C2 at release 3. Note that these are not mutually exclusive samples over time and different SNPs predict covid-19 each time. CI=confidence interval

In the no relevance setting (ie, testing the association with covid-19 as the exposure), with the exception of the association between covid-19 severity and BMI with release 3 (July 2020), the analyses were consistent with the no evidence of an effect throughout (fig 7). We would expect that the earliest data releases were the most selective because universal testing was not available in the early phase of the pandemic.

Our analysis shows the importance of replicating analyses over time, particularly when external factors could introduce bias differentially. Although we saw changes in the magnitude of the point estimates over time for the main analysis (BMI-covid-19), these changes likely reflected a heterogeneous population and increasing sample size over time. Taken together with the no relevance results over time (for which null results over time would indicate no evidence of selection bias), where we found evidence of an effect of covid-19 on BMI at the earliest release (when universal testing was not available), this finding likely reflects potential selection bias at the earliest data release. In this covid-19 example, however, with the data that are available, we acknowledge that comparing results across time is compromised by differences in power at different time points. For others using this approach with other research questions, having sufficiently large and similar sized samples at each time point would be valuable. The magnitude of differences with time that would indicate bias depends on the specific research question and the extent to which selection or exposure or outcome measurement error, or both, are likely to have changed over time. Similar changes in selection could be relevant for other conditions and MR analyses of these. For example, although not implemented in all countries, evidence exists for a drive to introduce universal testing for gestational diabetes, which could change who is diagnosed as a case. This change in testing would subsequently change the sample used in GWAS for gestational diabetes.

## Additional approaches for individual level MR analyses

In this paper, we have focused on the summary data MR approach. This approach is more commonly used in the literature because of the widespread availability of GWAS summary statistics and the greater power from the larger sample sizes available in these GWAS. When large enough studies with individual level data on genotypes, phenotypes, and disease outcomes exist, individual level MR can be conducted. These individual level data would support more methods for exploring or accounting for selection bias. For example, inverse probability weighting could be used to weight the study sample, based on predictors of selection, to better reflect the population of interest.^9^ Alternatively, the presence of potential biases could be evaluated with bias component plots, exploring the balance of variables predicting selection across levels of the instrument and outcome.^62^ Analysts would also have more power to detect whether pleiotropy is present by evaluating the correlations between covid-19 SNPs and the actual predictors of getting tested, such as education (ie, whether an association between covid-19 SNPs and reported education phenotypes exists), rather than using linkage disequilibrium score regression.

## Final remarks

We have systematically carried out a range of methods (box 2) to help test for selection and misclassification biases, with an illustrative example of BMI and covid-19. However, we wish to highlight that none of these methods are specifically designed to look at selection and misclassification bias. Although quantitative bias analyses in the non-MR literature exist, which have a similar principle,^63^ here we have used a range of methods to qualitatively assess the degree to which there might be bias, rather than asking how much bias would there need to be to make our results null? Knowledge of the subject matter is required to interpret the results and consider whether there might be other explanations for any differences in findings. Along with assessing the potential for selection and misclassification bias in MR, we evaluated the robustness of the main inverse variance weighted analysis to the three core MR assumptions. Other assumptions are made in MR, such as assuming the instrument acts in the same way in all individuals (ie, the monotonicity assumption, estimating the local average treatment effect); we have made these here. More discussion of these issues and methods to deal with them can be found in the literature.^36^ ^64^

Survival bias is another form of selection bias; participants are only included in analyses if they are (usually) healthy enough to do so. Although not discussed explicitly here, we encourage researchers to refer to the literature.^65^ ^66^ ^67^ Similarly, index event bias might also be present in MR analyses of disease progression of covid-19,^51^ which can occur when conditioning on an index event (ie, SARS-CoV-2 infection). Recently, methods have been developed to account for this bias in MR^67^ ^68^ and compared.^69^ MR also requires large sample sizes to obtain sufficient statistical power, and MR extensions, such as multivariable MR, require even larger sample sizes. Although summary data MR can be more efficient, inferences of results might still be limited by low power, and the application of these methods should be considered with this in mind.

Although this work was motivated by studies of determinants of covid-19 in its acute phase, these methods are likely to be relevant in exploring determinants of long covid and long term adverse outcomes in those who have had covid-19 disease. Selection of who is invited to join a study, responds to that invite, has all of their data collected, and remains in the study is ubiquitous in all human research. For many health outcomes, diagnosis of a case relies on characteristics that might be related to potential determinants of that disease explored in MR analyses. The effect of selection and misclassification bias depends on the variables included in an analysis and on the underlying data used. Thus we strongly recommend that researchers undertaking MR consider using the methods we propose here to explore bias caused by selection and misclassification. Depending on the available data, however, it might not be possible to conduct all of the analyses presented here.

## Data Availability

The datasets analysed during this study are publicly available genome wide association studies. Analysis scripts and the analysis plan can be found on this GitHub page: https://github.com/gc13313/BMI_COVID_2SMR.

## Web extra

Extra material supplied by authors

Web appendix: Supplementary figures and tables

## Notes

Contributors: GLC and AGS contributed equally and are joint first authors. DAL and ARC contributed equally and are joint last authors. DAL conceived the study. All authors designed the study. GLC wrote the analysis plan with input from all authors. AGS, NG, GLC, and ARC performed the analyses. GLC and ARC wrote the original draft of the manuscript with input from all authors. All authors were involved in the interpretation of results, helped refine the manuscript, and approved its final version. This publication is the work of the authors and GLC, AGS, NG, DAL, and ARC will serve as guarantors for the contents of this paper. The corresponding author attests that all listed authors meet authorship criteria and that no others meeting the criteria have been omitted. The manuscript’s guarantors affirm that the manuscript is an honest, accurate, and transparent account of the study being reported; that no important aspects of the study have been omitted; and that any discrepancies from the study as planned and registered have been explained.

Provenance and peer review: Not commissioned; externally peer reviewed.
