# Supplementary material for: A framework for assessing selection and misclassification bias in mendelian randomisation studies: an illustrative example between body mass index and covid-19
Source: BMJ. 2023 Jun 19;381:e072148. doi: 10.1136/bmj-2022-072148 (PMC10277657; doi:10.1136/bmj-2022-072148)
Supplement: Supplementary file 1 — Web appendix: Supplementary figures and tables [file clag072148.ww.pdf]

Supplementary figures and tables

Supplementary Table 1 Summary of Mendelian randomization methods and sensitivity analyses used
Supplementary Table 2 Summary of one/two sample Mendelian randomisation (MR) studies that assessed the effect of body mass index (BMI) on SARS-CoV-2/COVID-19
Supplementary Table 3 Studies contributing to the COVID-19 Host Genetics Initiative
Supplementary Figure 1 Flow chart of methods carried out to investigate selection and misclassification bias
Supplementary Figure 2 Flow chart of SNPs included in the main analysis of body mass index (BMI) on SARS-CoV-2/COVID-19
Supplementary Figure 3 (a) Main analysis: Effect of body mass index (BMI) on SARS-CoV-2/ COVID-19 (b) No-relevance analysis': Effect of SARS-CoV-2/COVID-19 on BMI
Supplementary Figure 4 Effect of body mass index (BMI) on SARS-CoV-2/COVID-19: Inverse variance weighting (IVW) estimates from most recent GIANT BMI GWAS that includes UK Biobank (UKB) and the previous GWAS that excludes UKB
Supplementary Table 4 Cochran's Q test results of between SNP heterogeneity for the main the Mendelian randomisation (MR) association of effect of body mass index (BMI) on COVID-19 (4a), and zero-relevance test of potential 'biased' effect of COVID-19 and BMI (4b)
Supplementary Table 5 MR Egger intercept results for the MR associations between body mass index (BMI) and COVID-19, and between COVID-19 and BMI
Supplementary Figure 5 Population stratification: (a) Effect of body mass index (BMI) on skin tanning; (b) Effect of COVID-19 on skin tanning
Supplementary Table 6 SNPs associated with COVID-19
Supplementary Table 7 Characteristics of genome-wide association studies used in the multivariable MR
Supplementary Figure 6. Forest plots of single SNP analysis of the association between COVID-19 and body mass index (BMI): for comparisons A1, A2 and B2 respectively (where there was some evidence of SNP heterogeneity)

Supplementary Table 1 Summary of Mendelian randomization methods and sensitivity analyses used

<table>
<tr><th>Method</th><th colspan="2">Description</th></tr>
<tr><td colspan="3">Main analyses</td></tr>
<tr><td>Mendelian randomisation (MR)</td><td colspan="2">An instrumental variable approach that uses genetic variants as instruments for a modifiable exposure. MR is less prone to bias by confounding and reverse causality due to the random allocation of genetic variants at conception. However, a number of strong, often unverifiable assumptions are required. Genetic instruments must 1) be robustly and statistically strongly associated with the risk factor (Relevance assumption), 2) be independent of confounders between it and the outcome (Independence assumption) and 3) only be associated with the outcome via their effect on the risk factor (Exclusion restriction assumption).</td></tr>
<tr><td>Inverse variance weighted (IVW)</td><td colspan="2">Estimates a causal effect of the exposure on the outcome by regressing the SNP-exposure association on the SNP-outcome association and constraining the intercept of the regression to zero. Assumes that there is no unbalanced horizontal pleiotropy.</td></tr>
<tr><td colspan="3">Methods to explore MR assumptions</td></tr>
<tr><td>F-statistic</td><td>Relevance assumption and weak instrument bias</td><td>In the two-sample summary data context and with uncorrelated genetic variants, the F-statistic for variant j can be approximated as F_j =beta^2 / se^2. Higher F-statistic values suggest lower risk of weak instrument bias.
Both samples should be from the same underlying population (e.g. if an outcome can only occur in one sex, such as uterine or prostate cancer, then the SNP-exposure data should ideally come from a sample that is sex specific).</td></tr>
<tr><td>MR-Egger</td><td>Horizontal pleiotropy</td><td>Identical to IVW, except that it does not constrain the regression line to go through zero. This means that the slope of the MR-Egger regression represents the estimate of the causal effect controlling for unbalanced horizontal pleiotropy. A non-null MR-Egger intercept provides statistical evidence of horizontal pleiotropy. MR-Egger has less statistical power than IVW and there should be no violation of the Instrument Strength Independent of Direct Effect (InSIDE) assumption.</td></tr>
<tr><td>Cochran's Q statistics</td><td>Horizontal pleiotropy</td><td>Tests for between-SNP heterogeneity, which can indicate that one or more of the SNPs have horizontal pleiotropic paths</td></tr>
<tr><td>Weighted median (WM)</td><td>Horizontal pleiotropy</td><td>Provides an unbiased estimate when up to 50% of the weight of SNPs used in the instrument violate the IV assumptions (i.e. if one strong SNP, or several weaker ones, contribute to >50% of</td></tr>
</table>

		the total association between all SNPs and the exposure and have horizontal pleiotropic paths the will be biased).
Sample definition/ underlying population	Confounding (via population stratification)	Confounding of SNP-outcome associations (Independence assumption) can occur where there is population substruction within the study. To minimise this bias, GWAS and MR analyses are often carried out using on restricted samples (e.g., only including participants of European ancestry).
Negative control exposure/outcome	Population stratification	To assess bias caused by population stratification, i.e. confounding by population, we used skin tanning as a negative control outcome to compare the association observed between BMI and COVID-19 with the association observed between BMI and skin tanning. Here, a negative control would be a variable that is not expected to be associated with the outcome but has the same underlying population structure. Evidence of an association when using the negative control outcome could indicate bias from population stratification. We similarly explored bias in the COVID-19 GWAS by using COVID-19 as the exposure and skin tanning as the outcome.
Independent GWAS samples	Sample overlap	If samples overlap in two sample MR, then features of two-sample MR, become similar to those of one-sample MR, particularly with substantial overlap. For example, weak instrument bias in two sample MR is expected to bias towards the null, whereas in one sample it is towards the confounded observational analysis result.

Supplementary Table 2 Summary of individual level/summary data sample Mendelian randomisation (MR) studies that assessed the effect of body mass index (BMI) on SARS-CoV-2/COVID-19

Author, year (PMID)	Type of MR	BMI GWAS	SARS-CoV-2/COVID-19 comparison groups	Sample sizes/ Release	Statistical analyses	Main results	Attempt to explore selection bias/ misclassification
Gao et al, 2022 (35031696)	Summary data	Yengo et al.	COVID-19 vs population (C2)<br><br>Hospitalised COVID-19 vs population (B2)<br><br>Very severe respiratory COVID-19 vs population (A2)	38,984 COVID-19 cases/ 1,644,784 controls<br><br>9,986 hospitalised COVID-19 cases/ 1,877,672 controls<br><br>5,101 very severe respiratory COVID-19 cases / 1,383,241 controls<br><br>Release 5 (18th January 2021)	Main: IVW<br>Sensitivity: MR-Egger, weighted median, weighted mode<br><br>Observational: UKB	OR 1.20 (95% CI 1.13, 1.28) per 1 SD increase in BMI (C2)<br><br>OR 1.50 (95% CI 1.32, 1.70) per 1 SD increase in BMI (B2)<br><br>OR 1.44 (95% CI 1.19, 1.73) per 1 SD increase in BMI (A2)	No
Leong et al, 2021 (33661905)	Summary data	Locke et al.	COVID-19 vs population (C2) – main analyis<br><br>Hospitalised COVID-19 vs population (B2) – main analysis<br><br>Very severe COVID-19 vs not hospitalised COVID-19 (A1)<br><br>Very severe COVID-19 vs poulation (A2)	14,134 COVID-19 cases/ 1,284,876 controls<br><br>6,406 hospitalised COVID-19 cases/ 902,088 controls<br><br>269 very severe COVID-19 cases/ 688 controls<br><br>4,933 very severe COVID-19 cases/ 1,398,672 controls	Main: IVW<br>Sensitivity: weighted median, MR-Egger, mode-base estimate, MR-PRESSO, leave-one-out analysis	OR 1.29 (95% CI 1.10, 1.53) per 1 SD increase in BMI (C2)<br><br>OR: 1.69 (95% CI 1.31, 2.17) per 1 SD increase in BMI (B2)<br><br>OR 1.04 (95%CI 0.24, 4.50) per 1 SD increase in BMI (A1)<br><br>OR 1.67 (95%CI 1.24, 2.26) per 1 SD increase in BMI (A2)	Comparison of different case/control groups but not looking specifically at selection/ misclassification

			Hospitalised COVID-19 vs not hospitalised COVID-19 (B1)<br><br>COVID-19 vs lab/self-reported COVID-19 negative (C1)<br><br>Predicted COVID-19 vs predicted or self-reported COVID-19 negative (D1)	2,430 hospitalised COVID-19 cases/ 8,488 controls<br><br>24,057 COVID-19 cases / 218,062 controls<br><br>3,204 COVID-19 cases/ 35,728 controls<br><br>Release 4 (20th October 2020)		OR 1.50 (95% CI 0.95, 2.37) per 1 SD increase in BMI (B1)<br><br>OR 1.21 (95% CI 1.02, 1.44) per 1 SD increase in BMI (C1)<br><br>OR (0.93, 95%CI 0.71, 1.21) per 1 SD increase in BMI (D1)	
Li S et al, 2021 (33536004)	Summary data	Yengo et al.	Very severe respiratory COVID-19 vs population (A2)<br><br>Hospitalised COVID-19 vs population (B2)	2,972 cases / 284,472 controls<br><br>6,492 cases / 1,012,809 controls<br><br>Release 4 (September 2020)	Main: IVW Sensitivity: MR-Egger, weighted median, weighted mode	OR 1.91 (95% CI 1.55, 2.35) per 1 SD increase in BMI (A2)<br><br>OR 1.75 (95% CI 1.52, 2.01) per 1 SD increase in BMI (B2)	No
Lorincz-Comi et al, 2021 (33846372)	Summary data	Yengo et al.	COVID-19 hospitalised vs not hospitalised COVID-19 (B1)	928 cases / 2,028 controls (EUR)<br><br>2,430 cases/ 8,378 controls (Mixed)<br><br>Release 3 for EUR (2nd July 2020) and 4 for mixed (20th October 2020)	Main: IVW Sensitivity: MRMix, IMRP, MR-Egger	OR 0.89 (95% CI 0.62, 1.28) per 1 SD increase in BMI (EUR)<br><br>OR 1.12 (95% CI 0.90, 1.38) per 1 SD increase in BMI (Mixed)	No
Freuer et al, 2021 (33631142)	Summary data	Pulit et al.	COVID-19 vs population (C2)	6,696 cases/ 1,073,072 controls)	Main: radial IVW, random-effects IVW	OR 1.31 (95%CI 1.15, 1.50) per 1 SD increase in BMI (C2)	No

			Hospitalised COVID-19 vs population (B2)	3,199 cases/ 897,488 controls<br><br>Release 3 (29th June 2020)	Sensitivity: MR-PRESSO, RAPS, weighted mean, weigted mode, MR-Egger	OR 1.62 (95% CI 1.32, 1.99) per 1 SD increase in BMI (B2)	
Ponsford et al, 2020 (32966752)	Summary data	Yengo et al.	Severe COVID-19 vs controls (with no or mild COVID-19 symptoms)<br><br>Hospitalised COVID-19 vs population (B2)	1,610 cases/ 2,205 controls in Italy and Spain<br><br>3,199 cases/ 897,488 controls<br><br>Release 3 (29th June 2020)	Main: IVW Sensitivity: MR-Egger, weighted median	OR: 1.75 (95% CI 1.20, 2.57) per 1 SD increase in BMI (severe COVID-19)<br><br>OR 1.47 (95% CI 1.18, 1.83) per 1 SD increase in BMI (B2)	
Aung et al, 2020 (33262790)	Individual level and summary data	Locke et al.	COVID-19 vs controls (tested negative or untested) in UK Biobank<br><br>COVID-19 vs population (C2)	1,211 cases / 387,079 controls 16th March 2020 – 31st May 2020<br><br>1,678 cases/ 674,635 controls<br><br>Release 2 (15th May 2020)	Main: IVW Sensitivity: MR-Egger, weighted median, MR-PRESSO	OR 1.15 (1.05, 1.36) per 1 SD increase in BMI (UKB)<br><br>OR 1.80 (95% CI 1.28, 2.54) per 1 SD increase in BMI (C2)	Sensitivity analysis in 1SMR restricted to only those tested for SARS-CoV-2

EUR: Europeans; IMRP: Iterative Mendelian Randomization and Pleiotropy approach; IVW: inverse-variance weighting, MRMix: Mendelian randomisation using mixture models; MR-PRESSO: Mendelian Randomisation Pleiotropy RESidual Sum and Outlier

Supplementary Table 3 Studies contributing to the COVID-19 Host Genetics Initiative

Group/studies	Cases	Controls
**A1: Very severe respiratory confirmed COVID-19 vs not hospitalised COVID-19**		
BQC19_EUR	55	151
BoSCO_EUR	59	262
FinnGen_FIN	54	224
SPGRX_EUR	101	51
**A2: Very severe respiratory confirmed COVID-19 vs population**		
ANCESTRY_Freeze_Four_EUR	667	113882
Amsterdam_UMC_COVID_study_group_EUR	66	1413
BQC19_EUR	165	743
BRACOVID_AMR	539	1149
BelCovid_EUR	184	1477
CHULACOVID_EAS	137	326
CU_AFR	133	2610
CU_EUR	203	2149
Egypt_hgCOVID_hub_AFR	167	180
FinnGen_FIN	190	278193
GENCOVID_EUR	1077	2443
GHS_Freeze_145_EUR	321	108168
GNH_SAS	151	34013
HOSTAGE_EUR	1779	9116
INMUNGEN_CoV2_EUR	70	654
JapanTaskForce_EAS	155	1705
PMBB_AFR	55	8622
SPGRX_EUR	101	302
SaudiHumanGenomeProgram_ARAB	147	768
SweCovid_EUR	113	3748
UKBB_EUR	417	420114
Vanda_EUR	58	900
genomicc_EAS	149	745
genomicc_EUR	1676	8380
idipaz24genetics_EUR	59	75
**B1: Hospitalised COVID-19 vs not hospitalised COVID-19**		
ANCESTRY_Freeze_Four_AFR	135	1494
ANCESTRY_Freeze_Four_AMR	199	3553
ANCESTRY_Freeze_Four_EUR	1484	23869
BelCovid_EUR	365	121
BoSCO_EUR	212	512
CHOP_CAG_AFR	147	412
DECODE_EUR	89	1808
EstBB_EUR	271	2335
FHoGID_EUR	470	280
GENCOVID_EUR	1267	331
GHS_Freeze_145_EUR	773	4503
GNH_SAS	339	4812

Generation_Scotland_EUR	554	3674
IranCovid_MID	210	183
MVP_AFR	1300	3573
MVP_AMR	517	1962
MVP_EUR	2417	9251
PMBB_AFR	66	100
QGP_ARAB	60	640
SPGRX_EUR	311	51
UKBB_AFR	101	291
UKBB_CSA	126	489
UKBB_EUR	3067	8947
**B2: Hospitalised COVID-19 vs population**		
ANCESTRY_Freeze_Four_EUR	1484	113882
Amsterdam_UMC_COVID_study_group_EUR	108	1413
BQC19_EUR	378	530
BRACOVID_AMR	853	835
BelCovid_EUR	367	1477
CHULACOVID_EAS	137	326
CU_AFR	304	2610
CU_EUR	453	2149
Egypt_hgCOVID_hub_AFR	167	180
FinnGen_FIN	273	278193
GENCOVID_EUR	1287	2443
GHS_Freeze_145_EUR	773	108168
GNH_SAS	339	33825
HOSTAGE_EUR	3151	12483
JapanTaskForce_EAS	572	1705
PMBB_AFR	126	8551
SPGRX_EUR	311	302
SaudiHumanGenomeProgram_ARAB	147	768
SweCovid_EUR	113	3748
UKBB_EUR	3067	417464
Vanda_EUR	58	900
genomicc_EAS	149	745
genomicc_EUR	1676	8380
idipaz24genetics_EUR	106	75
**C1: COVID-19 vs lab/self-reported negative**		
23ANDME_AFR	506	3110
23ANDME_EUR	9913	85072
23ANDME_HIS	2553	13086
ANCESTRY_Freeze_Four_EUR	2417	14933
BQC19_EUR	206	327
DECODE_EUR	1897	29014
EstBB_EUR	313	12019
GNH_SAS	114	256
INTERVAL_EUR	161	1119
Lifelines_EUR	358	1253
MGI_EUR	122	508

MVP_AFR	1217	9204
MVP_AMR	510	3325
MVP_EUR	1520	24135
NTR_EUR	145	117
PHBB_AFR	60	375
PHBB_EUR	151	3118
PHBB_HIS	66	276
PMBB_AFR	166	934
UKBB_AFR	68	268
UKBB_CSA	66	350
UKBB_EUR	1310	13646
genomicsengland100kgp_EUR	218	1617
**C2: COVID-19 vs population**		
ACCOuNT_AFR	57	799
ANCESTRY_Freeze_Four_AFR	1629	5688
ANCESTRY_Freeze_Four_AMR	3752	12323
ANCESTRY_Freeze_Four_EAS	120	570
ANCESTRY_Freeze_Four_EUR	25353	113882
Amsterdam_UMC_COVID_study_group_EUR	108	1413
BQC19_EUR	477	431
BRACOVID_AMR	853	835
BelCovid_EUR	488	1477
BioVU_EUR	141	70615
CCHC_AMR_HIS	95	3140
CCPM_AMR	220	3111
CCPM_EUR	896	24851
CHULACOVID_EAS	137	326
CU_AFR	332	2610
CU_EUR	508	2149
Corea_EAS	108	6500
Coronagenes_EUR	181	734
DECODE_EUR	4256	270934
EXCEED_EUR	236	1083
Egypt_hgCOVID_hub_AFR	167	180
EraCORE_EUR	302	1646
EstBB_EUR	4070	189406
FinnGen_FIN	2695	278193
GCAT_EUR	447	4541
GENCOVID_EUR	1697	2443
GFG_EUR	136	4978
GHS_Freeze_145_AFR	128	3050
GHS_Freeze_145_AMR	88	1307
GHS_Freeze_145_EUR	5276	108168
GNH_SAS	5151	29013
Generation_Scotland_EUR	1712	17525
Genetics_COVID19_Korea_EAS	624	6549
Genotek_EUR	676	12317
HOSTAGE_EUR	3151	12483

HUNT_EUR	283	52072
Helix_EUR	178	5441
INMUNGEN_CoV2_EUR	236	654
INTERVAL_EUR	2098	39733
JapanTaskForce_EAS	614	1705
LGDB_EUR	275	1313
Lifelines_EUR	895	26600
MGI_AFR	111	2989
MGI_EUR	122	51458
MOBA_EUR	391	56038
MVP_AFR	4893	94556
MVP_AMR	2497	41100
MVP_EUR	11778	357198
NTR_EUR	228	5265
PMBB_AFR	379	8298
PMBB_EUR	60	9702
QGP_ARAB	700	13360
SINAI_COVID_EUR	942	3784
SPGRX_EUR	362	302
SaudiHumanGenomeProgram_ARAB	147	768
Stanford_EUR	169	190
SweCovid_EUR	113	3748
TOPMed_CHRIS10K_EUR	92	2373
TOPMed_Gardena_EUR	452	458
TwinsUK_EUR	247	2320
UKBB_AFR	426	6210
UKBB_AMR	51	929
UKBB_CSA	695	8181
UKBB_EAS	104	2605
UKBB_EUR	13256	407275
UKBB_MID	89	1510
Vanda_EUR	58	900
WGHS_EUR	225	11887
genomicc_EAS	149	745
genomicc_EUR	1676	8380
genomicsengland100kgp_EUR	822	44369
genomicsengland100kgp_SAS	235	4205
idipaz24genetics_EUR	106	75
thaicovid_EAS	191	113
**D1: predicted COVID-19 from self-reported symptoms vs predicted or self-reported non COVID-19**		
GS_EUR	132	3610
GeneRISK_EUR	154	3451
Helix_EUR	605	4778
Lifelines_EUR	1427	16833
NTR_EUR	603	4669
RS_EUR	283	2387

Supplementary Figure 1 Flow chart of methods carried out to investigate selection and misclassification bias

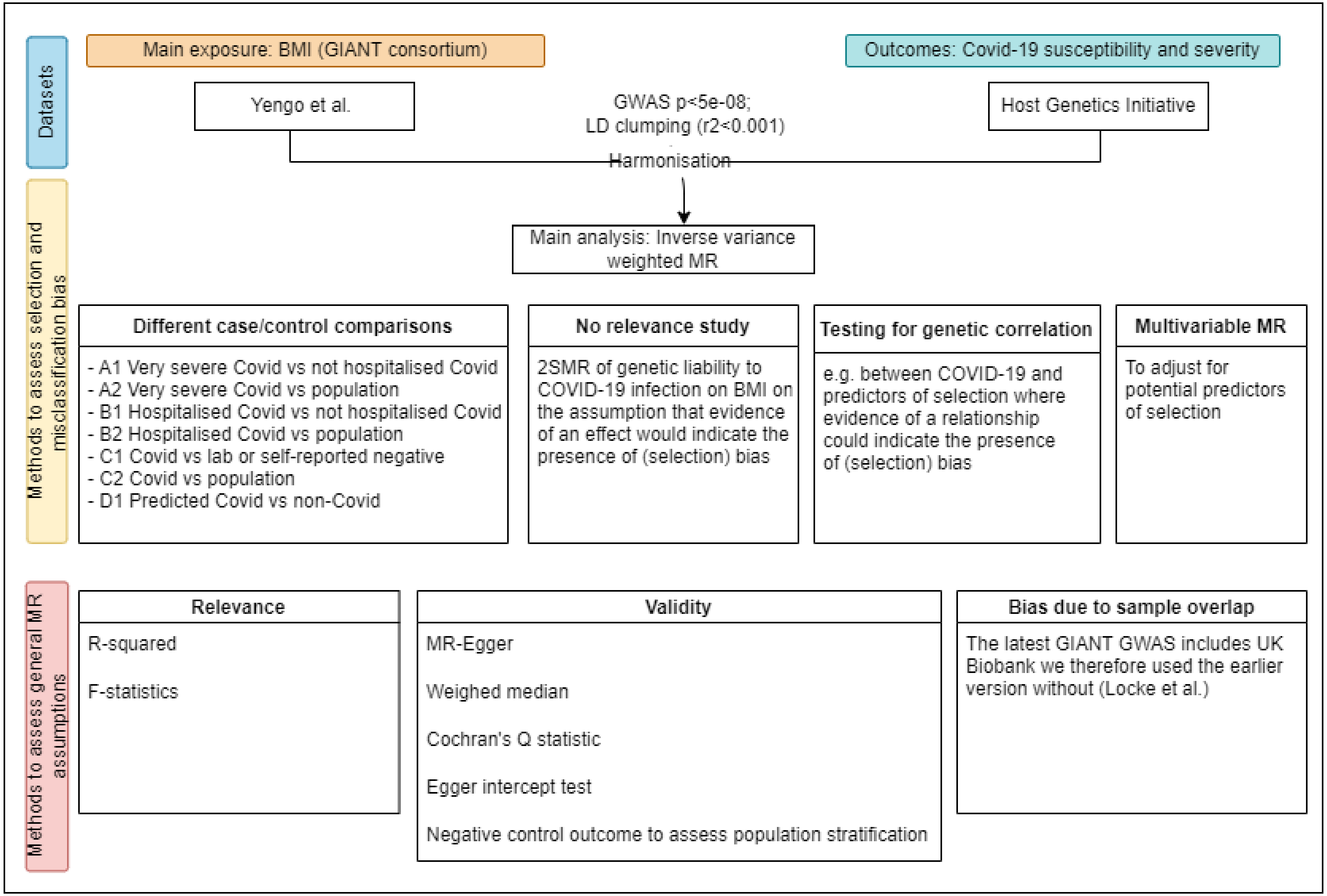

Supplementary Figure 2 Flow chart of SNPs included in the main analysis of body mass index (BMI) on SARS-CoV-2/COVID-19

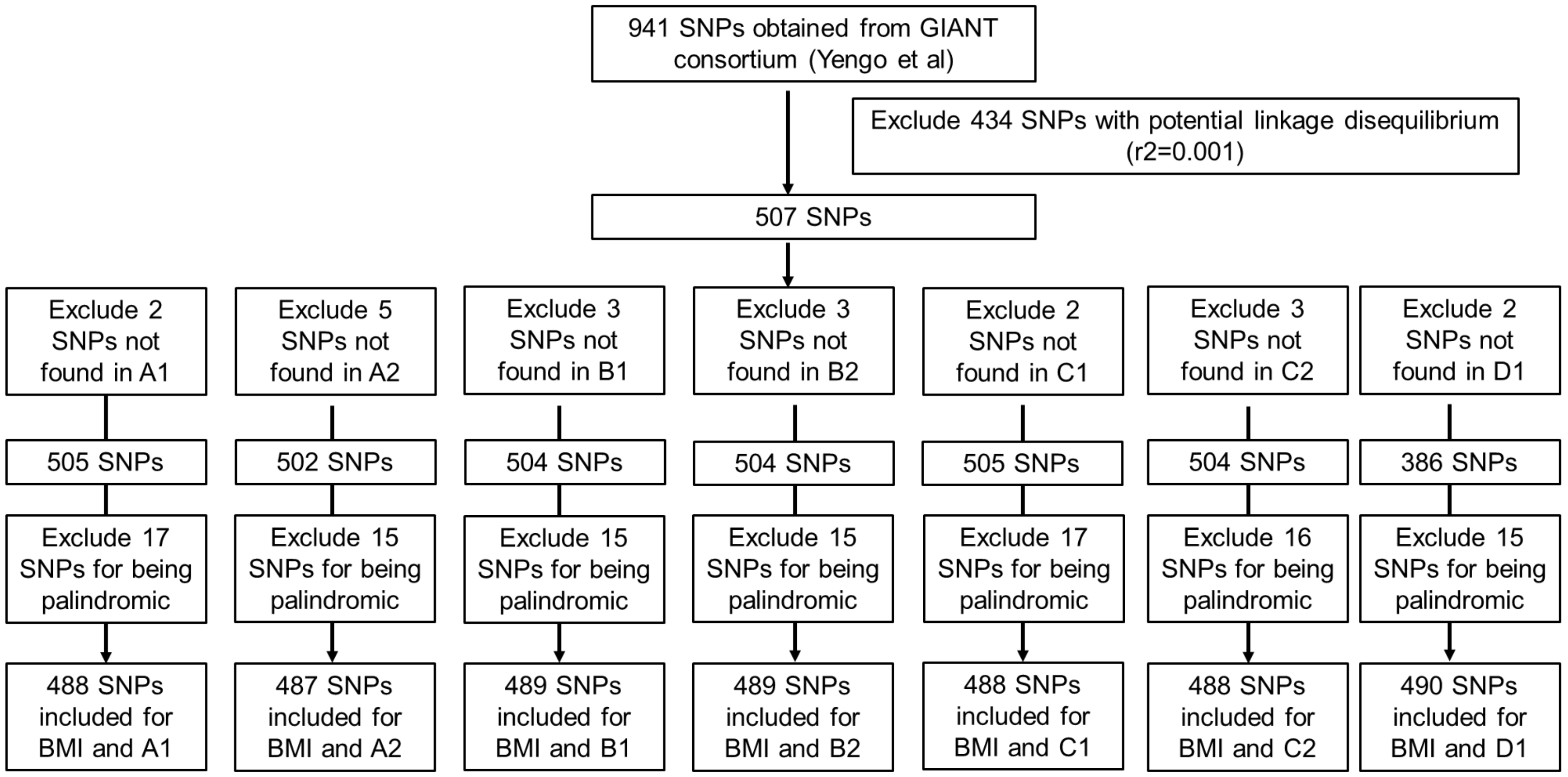

SNPs= single-nucleotide polymorphisms; GIANT=Genetic Investigation of ANthropometric Traits

A1: very severe respiratory confirmed COVID-19 vs not hospitalised COVID-19; A2: very severe respiratory confirmed COVID-19 vs population; B1: hospitalised COVID-19 vs not hospitalised COVID-19; B2: hospitalised COVID-19 vs population; C1: COVID-19 vs lab/self-reported negative; C2: COVID-19 vs population; D1: predicted COVID-19 from self-reported symptoms vs predicted or self-reported non COVID-19.
For comparisons A1, C1 and D1, one proxy SNP was included.

Supplementary Figure 3 (a) Main analysis: Effect of body mass index (BMI) on SARS-CoV-2/ COVID-19 (b) No-relevance analysis': Effect of SARS-CoV-2/COVID-19 on BMI

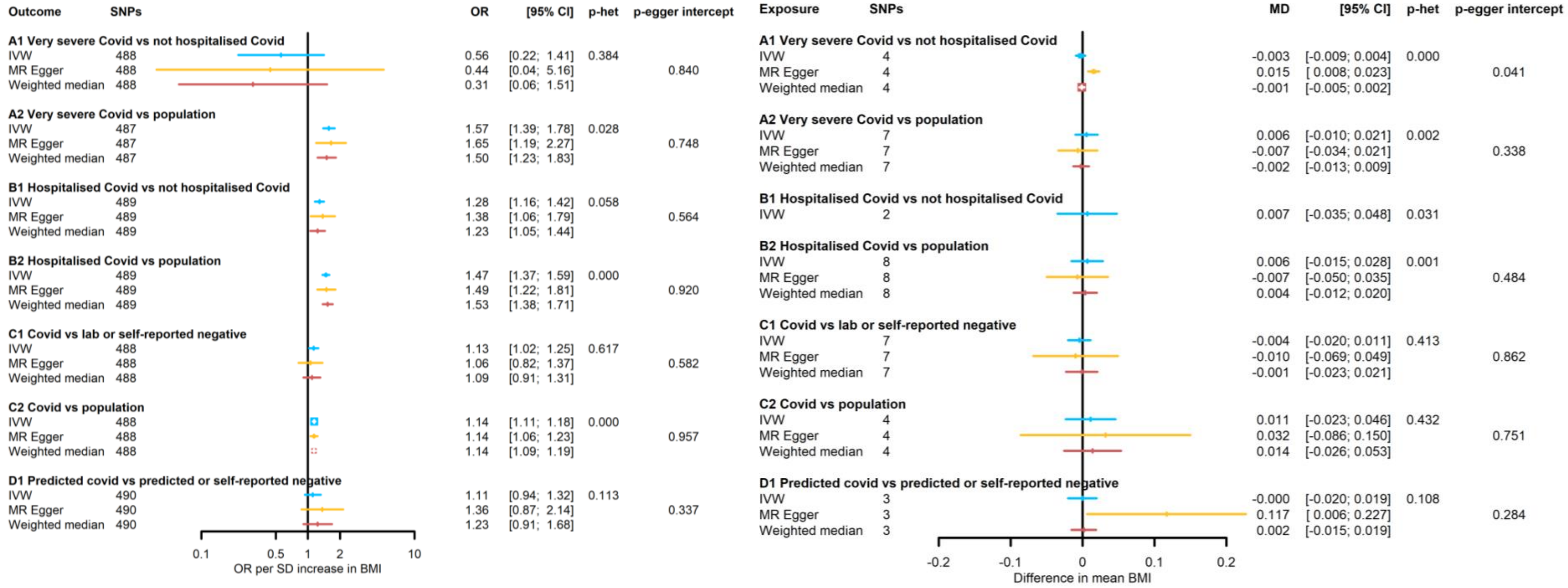

Footnote: IVW: inverse variance weighting; SNPs: single nuclear polymorphism. Groups A1, C1 and D1 are from version 4 whilst the others are from version 6. Note that different studies are contributing to each case/control group (see **Supplementary Table 2**), p-het: p-value from Cochranes Q statistic assessing SNP heterogeneity (see **Supplementary Table 4** for Cochranes Q statistic); p-egger intercept: p-value for MR Egger intercept (see **Supplementary Table 5** for intercept and standard error). For the 'no relevance' analysis, for groups A1, C1 and D1 a threshold of $p<5x10^{-6}$ was used for selection of SNPs; C1 includes one proxy SNP, and D1 includes 2 proxy SNPs.

Supplementary Figure 4 Effect of body mass index (BMI) on SARS-CoV-2/COVID-19: Inverse variance weighting (IVW) estimates from most recent GIANT BMI GWAS that includes UK Biobank (UKB) and the previous GWAS that excludes UKB

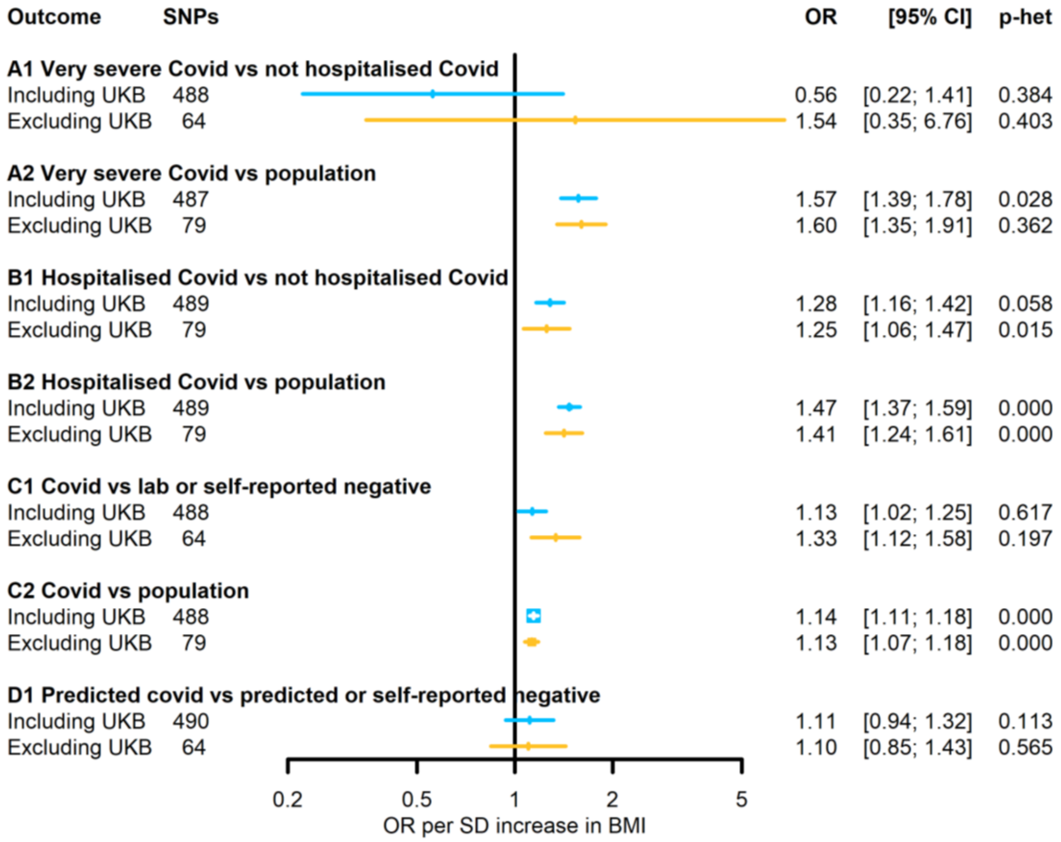

SNPs: single nuclear polymorphism, p-het: p-value from Cochranes Q statistic assessing SNP heterogeneity

Supplementary Table 4 Cochran's Q test results of between SNP heterogeneity for the main the Mendelian randomisation (MR) association of effect of body mass index (BMI) on COVID-19 (4a), and zero-relevance test of potential 'biased' effect of COVID-19 and BMI (4b)

Covid group	Method	Q test	df	p-value
**4a Main analyses (BMI on COVID-19)**				
A1: Very severe COVID-19 vs not hospitalised COVID-19	IVW	495.6	487	0.384
	MR Egger	495.5	486	0.373
A2: Very severe COVID-19 vs population	IVW	547.2	486	0.028
	MR Egger	547.1	485	0.026
B1: Hospitalised COVID-19 vs not hospitalised COVID-19	IVW	538.1	488	0.058
	MR Egger	537.7	487	0.055
B2: Hospitalised COVID-19 vs population	IVW	608.7	488	1.56E-04
	MR Egger	608.7	487	1.38E-04
C1: COVID-19 vs lab or self-reported negative	IVW	477.1	487	0.617
	MR Egger	476.8	486	0.609
C2: COVID-19 vs population	IVW	618.8	487	4.56E-05
	MR Egger	618.8	486	4.02E-05
D1: Predicted COVID-19 vs predicted or self-reported	IVW	527.1	489	0.113
negative	MR Egger	526.1	488	0.113
**4b 'No-relevance analyses' (MR of COVID-19 on BMI)**				
A1: Very severe COVID-19 vs not hospitalised COVID-19	IVW	23.2	3	<0.001
	MR Egger	0.2	2	0.890
A2: Very severe COVID-19 vs population	IVW	21.4	6	0.002
	MR Egger	17.5	5	0.004
B1: Hospitalised COVID-19 vs not hospitalised COVID-19	IVW	4.7	1	0.031
B2: Hospitalised COVID-19 vs population	IVW	23.9	7	0.001
	MR Egger	21.9	6	0.001
C1: COVID-19 vs lab or self-reported negative	IVW	6.1	6	0.413
	MR Egger	6.1	5	0.301
C2: COVID-19 vs population	IVW	2.8	3	0.432
	MR Egger	2.6	2	0.275
D1: Predicted COVID-19 vs predicted or self-reported	IVW			
negative		4.5	2	0.108
	MR Egger	0.1	1	0.790

Supplementary Table 5 MR Egger intercept results for the MR associations between body mass index (BMI) and COVID-19, and between COVID-19 and BMI

Description	MR Egger intercept	SE	p-value
**Main analysis (MR of BMI on COVID-19)**			
A1: Very severe COVID-19 vs not hospitalised COVID-19	0.004	0.020	0.840
A2: Very severe COVID-19 vs population	-0.001	0.003	0.748
B1: Hospitalised COVID-19 vs not hospitalised COVID-19	-0.001	0.002	0.564
B2: Hospitalised COVID-19 vs population	0.000	0.002	0.920
C1: COVID-19 vs lab or self-reported negative	0.001	0.002	0.582
C2: COVID-19 vs population	0.000	0.001	0.957
D1: Predicted COVID-19 vs predicted or self-reported negative	-0.004	0.004	0.337
**'No-relevance analysis' (MR of COVID-19 on BMI)**			
A1: Very severe COVID-19 vs not hospitalised COVID-19	-0.022	0.005	0.041
A2: Very severe COVID-19 vs population	0.003	0.003	0.338
B2: Hospitalised COVID-19 vs population	0.002	0.003	0.484
C1: COVID-19 vs lab or self-reported negative	-0.002	0.003	0.575
C2: COVID-19 vs population	-0.001	0.003	0.751
D1: Predicted COVID-19 vs predicted or self-reported negative	-0.023	0.011	0.284

Note: it was not possible to estimate MR Egger intercept for group B1 in the 'no-relevance' analysis due to the small number of instruments (less than 3)

Supplementary Figure 5 Population stratification: (a) Effect of body mass index (BMI) on skin tanning; (b) Effect of COVID-19 on skin tanning

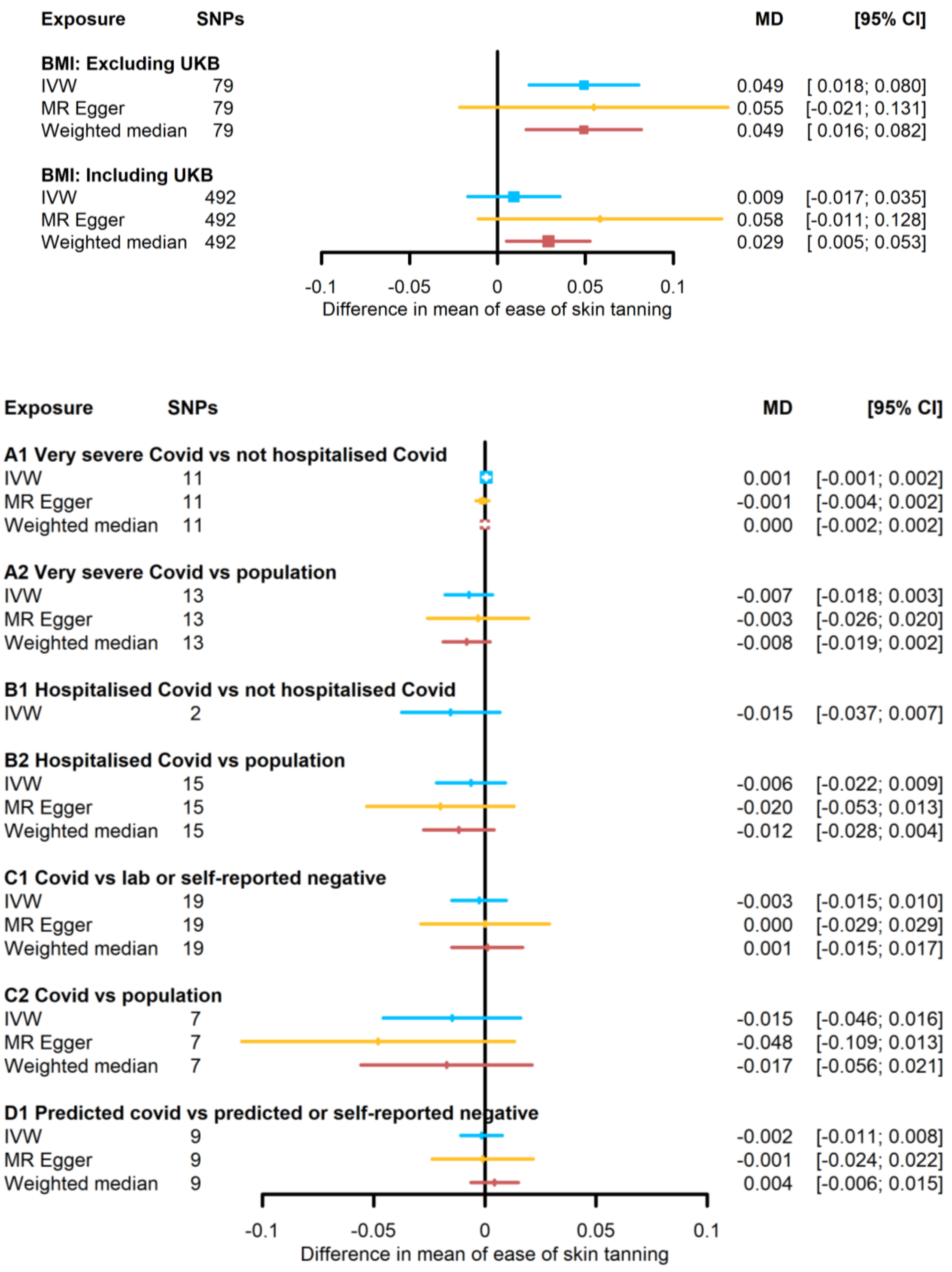

IVW: inverse variance weighting; SNPs: single nuclear polymorphism, MD: Mean difference (also referred to as Difference in mean); CI: Confidence interval

Supplementary Table 6 SNPs associated with COVID-19

Covid group	Sample size	rsID	effect allele	other allele	EAF	logOR	SE	P-value	Pseudo F-statistics	R^2
A1: Very severe Covid vs not hospitalised Covid	957	rs145285136	A	G	0.016	4.147	0.853	1.163E-06	23.6	0.024
	957	rs12069768	T	C	0.040	1.838	0.394	3.101E-06	21.8	0.022
	957	rs4462982	C	T	0.542	0.731	0.154	2.081E-06	22.5	0.023
	957	rs545631184	T	C	0.072	2.362	0.508	3.304E-06	21.6	0.022
	957	rs73064425	T	C	0.110	1.406	0.297	2.213E-06	22.4	0.023
	957	rs1107783	C	T	0.261	-0.889	0.193	4.284E-06	21.1	0.022
	957	rs12206024	A	G	0.188	0.896	0.194	3.916E-06	21.3	0.022
	957	rs59492037	G	A	0.100	1.283	0.259	7.072E-07	24.6	0.025
	957	rs112338868	C	T	0.016	4.242	0.838	4.114E-07	25.6	0.026
	957	rs1522633	A	T	0.375	0.809	0.157	2.76E-07	26.4	0.027
	957	rs3812875	G	A	0.037	1.771	0.381	3.437E-06	21.6	0.022
Harmonised SNPs									23.8	0.1059
When assuming a prevalence of 8%, total R^2=0.14										
A2: Very severe Covid vs population	1,010,654	rs10774671	A	G	0.668	0.126	0.019	3.60E-11	43.8	4.34E-05
	1,010,191	rs111837807	C	T	0.09793	0.204	0.03	2.15E-11	44.8	4.44E-05
	996,978	rs12259804	C	T	0.07149	-0.275	0.05	4.87E-08	29.8	2.99E-05
	992,464	rs12660421	A	G	0.03706	0.355	0.058	1.14E-09	37.1	3.73E-05
	1,009,233	rs13050728	C	T	0.6563	-0.167	0.02	1.24E-16	68.5	6.79E-05
	1,010,654	rs1405655	C	T	0.3229	0.114	0.019	4.06E-09	34.6	3.42E-05
	975,766	rs2277732	A	C	0.3153	0.203	0.021	8.48E-23	96.6	9.90E-05
	959,347	rs34536443	C	G	0.03966	0.394	0.06	4.89E-11	43.2	4.51E-05
	1,009,297	rs35154152	C	T	0.08958	-0.205	0.031	2.36E-11	44.7	4.42E-05
	1,009,760	rs35731912	T	C	0.08046	0.588	0.031	4.25E-81	363.8	3.60E-04
	1,010,251	rs495828	G	T	0.7857	-0.134	0.022	6.56E-10	38.1	3.78E-05
	996,635	rs71479671	A	G	0.4598	-0.131	0.024	4.19E-08	30.1	3.02E-05
	1,007,437	rs8080583	A	C	0.1789	-0.127	0.023	2.08E-08	31.4	3.12E-05

Harmonised SNPs									88.0	0.0006
When assuming a prevalence of 4%, total R^2=0.015										
B1: Hospitalised Covid vs not hospitalised Covid	87,671	rs11088247	G	C	0.3304	0.095	0.016	6.31E-09	33.7	3.85E-04
	87,505	rs17713054	A	G	0.09075	0.304	0.028	2.04E-28	122.2	0.0014
Harmonised SNPs									78.0	0.0018
When assuming a prevalence of 10%, total R^2=0.0028										
B2: Hospitalised Covid vs population	2,085,803	rs10774679	T	C	0.6505	0.076	0.011	4.70E-12	47.8	2.29E-05
	2,071,920	rs111837807	C	T	0.08627	0.113	0.018	9.46E-10	37.4	1.81E-05
	2,078,167	rs11702230	T	C	0.1174	0.1	0.017	3.22E-09	35.0	1.69E-05
	2,084,590	rs12809318	C	T	0.52	-0.063	0.011	9.53E-09	32.9	1.58E-05
	2,083,632	rs13050728	C	T	0.6568	-0.107	0.011	4.07E-21	88.9	4.27E-05
	2,085,803	rs1405655	C	T	0.3324	0.075	0.011	1.12E-11	46.1	2.21E-05
	2,050,749	rs2277732	A	C	0.2932	0.117	0.012	6.19E-22	92.7	4.52E-05
	2,019,040	rs34536443	C	G	0.03888	0.174	0.031	1.82E-08	31.7	1.57E-05
	2,071,167	rs35508621	C	T	0.07643	0.389	0.019	6.09E-89	399.8	1.93E-04
	1,790,208	rs35705950	T	G	0.09959	-0.112	0.02	1.22E-08	32.4	1.81E-05
	2,085,803	rs505922	T	C	0.6534	-0.1	0.011	2.09E-20	85.7	4.11E-05
	1,794,016	rs61667602	C	T	0.1743	-0.094	0.014	9.13E-12	46.5	2.59E-05
	2,084,446	rs67579710	A	G	0.1057	-0.103	0.017	1.03E-09	37.3	1.79E-05
	2,072,069	rs766826	T	C	0.3239	-0.082	0.012	1.15E-11	46.0	2.22E-05
	1,905,431	rs9367106	C	G	0.03992	0.202	0.031	3.40E-11	43.9	2.31E-05
Harmonised SNPs									99.2	0.0004
When assuming a prevalence of 3%, total R^2=0.0067										
C1: Covid vs lab or self-reported negative	110624	rs7535387	G	C	0.113	0.350	0.076	4.599E-06	21.0	0.000
	110624	rs4656878	A	G	0.470	0.095	0.021	3.907E-06	21.3	0.000
	110624	rs73062394	T	A	0.106	0.230	0.036	1.876E-10	40.6	0.000
	110624	rs144636229	G	A	0.081	0.320	0.070	4.683E-06	21.0	0.000
	110624	rs4974756	C	G	0.401	-0.099	0.022	4.084E-06	21.2	0.000
	110624	rs116335024	T	C	0.079	0.346	0.073	2.291E-06	22.3	0.000
	110624	rs4130109	A	T	0.800	-0.109	0.022	8.477E-07	24.2	0.000
	110624	rs72691535	A	C	0.112	0.173	0.034	3.055E-07	26.2	0.000

	110624	rs10000811	C	T	0.400	-0.079	0.016	7.644E-07	24.4	0.000
	110624	rs75309214	G	A	0.099	0.202	0.043	2.219E-06	22.4	0.000
	110624	rs9462875	G	A	0.235	0.096	0.019	6.5779E-07	24.7	0.000
	110624	rs2718228	A	C	0.540	0.105	0.021	5.173E-07	25.2	0.000
	110624	rs149487444	C	T	0.130	0.140	0.031	4.644E-06	21.0	0.000
	110624	rs75933622	A	G	0.089	-0.269	0.057	2.565E-06	22.1	0.000
	110624	rs147942333	A	T	0.075	0.415	0.080	2.168E-07	26.9	0.000
	110624	rs78101397	C	A	0.025	0.246	0.051	1.548E-06	23.1	0.000
	110624	rs436772	T	C	0.854	0.151	0.032	2.247E-06	22.4	0.000
	110624	rs144495330	G	A	0.081	0.297	0.063	2.682E-06	22.0	0.000
Harmonised SNPs									23.9	0.0012
	When assuming a prevalence of 10%, total R^2=0.0051 (including 1 proxy SNP)									
C2: Covid vs population	2,321,963	rs148063273	T	C	0.008178	0.13	0.023	8.47E-09	33.2	1.43E-05
	2,544,875	rs2109069	A	G	0.3084	0.033	0.005	1.68E-12	49.8	1.96E-05
	2,572,016	rs34288077	G	A	0.07597	0.077	0.008	1.17E-23	100.5	3.91E-05
	2,579,055	rs4342086	A	G	0.3399	-0.04	0.004	5.59E-20	83.8	3.25E-05
	2,513,309	rs4801778	T	G	0.174	-0.035	0.005	1.52E-10	41	1.63E-05
	2,586,332	rs505922	T	C	0.6505	-0.074	0.004	8.64E-68	302.7	1.17E-04
	2,234,283	rs73062389	A	G	0.04899	0.162	0.009	1.54E-65	292.3	1.3 E-04
Harmonised SNPs									134.2	0.0002
	When assuming a prevalence of 5%, total R^2=0.00069									
D1: Predicted Covid vs predicted or self-reported negative	38932	rs10926010	G	A	0.189	-0.170	0.036	2.762E-06	22.0	0.001
	38932	rs144997841	A	G	0.026	0.413	0.087	2.253E-06	22.4	0.001
	38932	rs146540521	T	C	0.012	0.658	0.136	1.212E-06	23.6	0.001
	38932	rs115480835	A	G	0.029	0.513	0.110	3.141E-06	21.7	0.001
	38932	rs13161855	A	G	0.135	-0.189	0.041	3.145E-06	21.7	0.001
	38932	rs11952066	T	C	0.788	-0.224	0.049	4.447E-06	21.1	0.001
	38932	rs72799046	A	G	0.058	0.319	0.064	6.3971E-07	24.8	0.001
	38932	rs144932426	A	G	0.013	0.691	0.145	1.871E-06	22.7	0.001

	38932	rs12972001	G	A	0.141	-0.194	0.042	3.703E-06	21.4	0.001
Harmonised SNPs									21.7	0.0006
When assuming a prevalence of 10%, total R^2=0.005 (including 2 proxy SNPs)										

EAF: effect allele frequency; SE: standard error
SNPs included after data harmonisation with BMI summary data are highlighted in grey
Note: p-value threshold for instruments was reduced to p< 5 x 10^{-6} for groups A1, C1 and D1

Supplementary Table 7 Characteristics of genome-wide association studies used in the multivariable MR

Exposure	PMID	DOI	N Sample size	N SNPs	% European	Unit	Covariable adjustments	Imputation panel
Education	3003 8396	10.1038/s41588-018-0147-3	766,345	10,101,24 2	100	years of schooling	Principal components Study-specific covariables	1000 Genomes at a minimum
Smoking	3064 3251	10.1038/s41588-018-0307-5	337,334	11,913,71 2	100	cigarettes per day	Principal components Study-specific covariables	1000 Genomes at a minimum
Income	NA	NA	397,751	9,851,867	100	SD	Principal components Study-specific covariables	1000 Genomes at a minimum

Supplementary Figure 6 Forest plots of single SNP analysis of the association between COVID-19 and body mass index (BMI): for comparisons A1, A2 and B2 respectively (where there was some evidence of SNP heterogeneity)

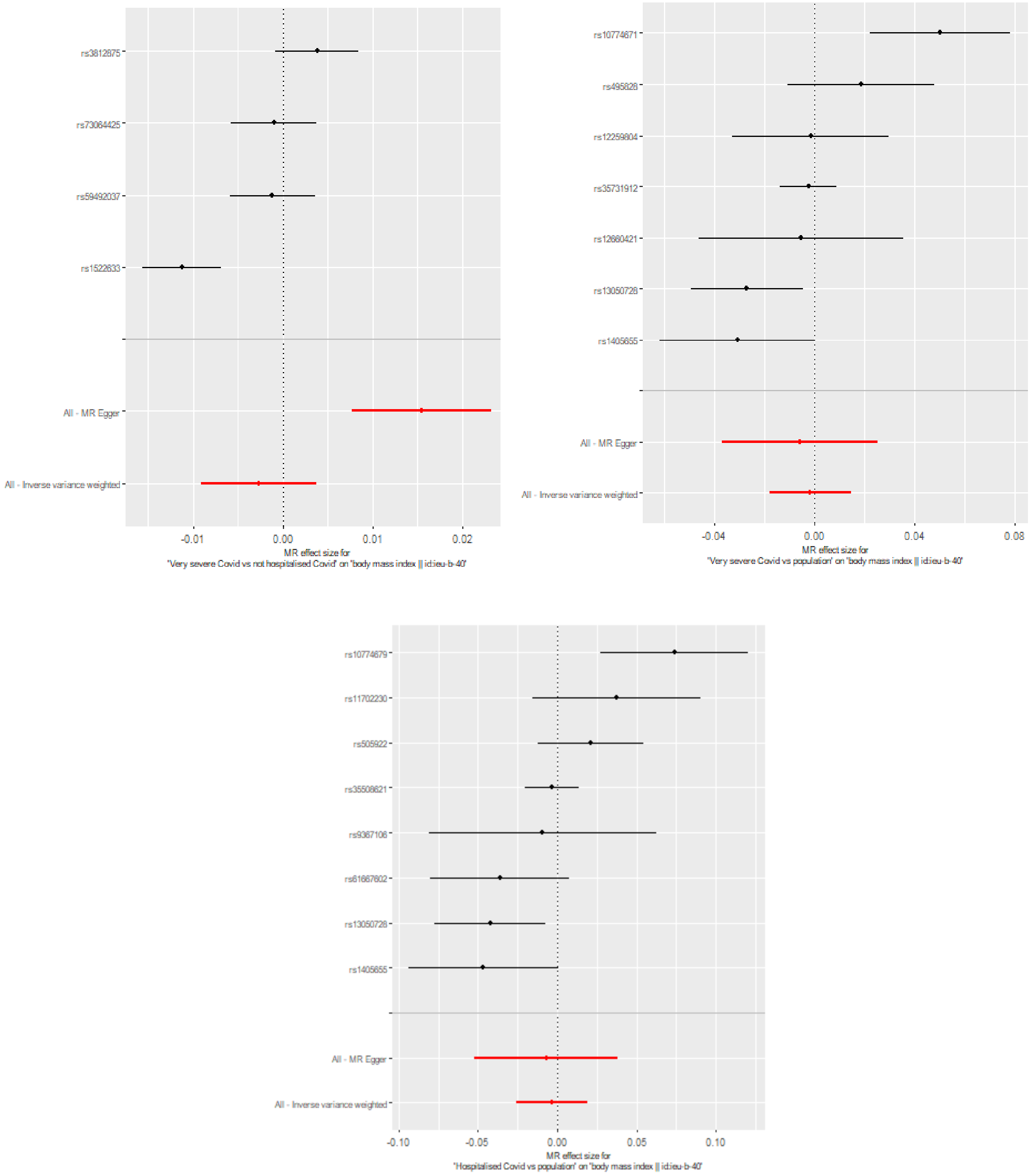

A1: very severe respiratory confirmed COVID-19 vs not hospitalised COVID-19; A2: very severe respiratory confirmed COVID-19 vs population; B2: hospitalised COVID-19 vs population
